# Chromosomal synapsis defects can trigger oocyte apoptosis without elevating numbers of persistent DNA breaks above wild-type levels

**DOI:** 10.1093/nar/gkac355

**Published:** 2022-05-17

**Authors:** Ramya Ravindranathan, Kavya Raveendran, Frantzeskos Papanikos, Pedro A San-Segundo, Attila Tóth

**Affiliations:** Institute of Physiological Chemistry, Faculty of Medicine, Technische Universität Dresden, Fetscherstraße 74, 01307 Dresden, Germany; Institute of Physiological Chemistry, Faculty of Medicine, Technische Universität Dresden, Fetscherstraße 74, 01307 Dresden, Germany; Institute of Physiological Chemistry, Faculty of Medicine, Technische Universität Dresden, Fetscherstraße 74, 01307 Dresden, Germany; Instituto de Biología Funcional y Genómica (IBFG), Consejo Superior de Investigaciones Científicas (CSIC) and University of Salamanca, Salamanca, Spain; Institute of Physiological Chemistry, Faculty of Medicine, Technische Universität Dresden, Fetscherstraße 74, 01307 Dresden, Germany

## Abstract

Generation of haploid gametes depends on a modified version of homologous recombination in meiosis. Meiotic recombination is initiated by single-stranded DNA (ssDNA) ends originating from programmed DNA double-stranded breaks (DSBs) that are generated by the topoisomerase-related SPO11 enzyme. Meiotic recombination involves chromosomal synapsis, which enhances recombination-mediated DSB repair, and thus, crucially contributes to genome maintenance in meiocytes. Synapsis defects induce oocyte apoptosis ostensibly due to unrepaired DSBs that persist in asynaptic chromosomes. In mice, SPO11-deficient oocytes feature asynapsis, apoptosis and, surprisingly, numerous foci of the ssDNA-binding recombinase RAD51, indicative of DSBs of unknown origin. Hence, asynapsis is suggested to trigger apoptosis due to inefficient DSB repair even in mutants that lack programmed DSBs. By directly detecting ssDNAs, we discovered that RAD51 is an unreliable marker for DSBs in oocytes. Further, SPO11-deficient oocytes have fewer persistent ssDNAs than wild-type oocytes. These observations suggest that oocyte quality is safeguarded in mammals by a synapsis surveillance mechanism that can operate without persistent ssDNAs.

## INTRODUCTION

Generation of haploid cells from diploid progenitors requires unique features of chromosome biology in meiosis. One of these features is the programmed formation of DNA double-stranded breaks (DSBs) at meiosis onset by a topoisomerase-like enzyme complex that consists of SPO11 and its binding partner TOPOVIBL ((1–5) and reviewed in (6,7)). Repair of DSBs by recombination generates reciprocal DNA exchanges, called crossovers, between homologous copies of each chromosome (homologs) in the first meiotic prophase. These inter-homolog crossovers enable correct meiotic chromosome segregation in most taxa including mammals. Maintenance of genome integrity requires that programmed DSBs are repaired before meiotic prophase exit, and that DSB repair results in the linkage of each homolog pair by crossovers.

**Figure 1.**
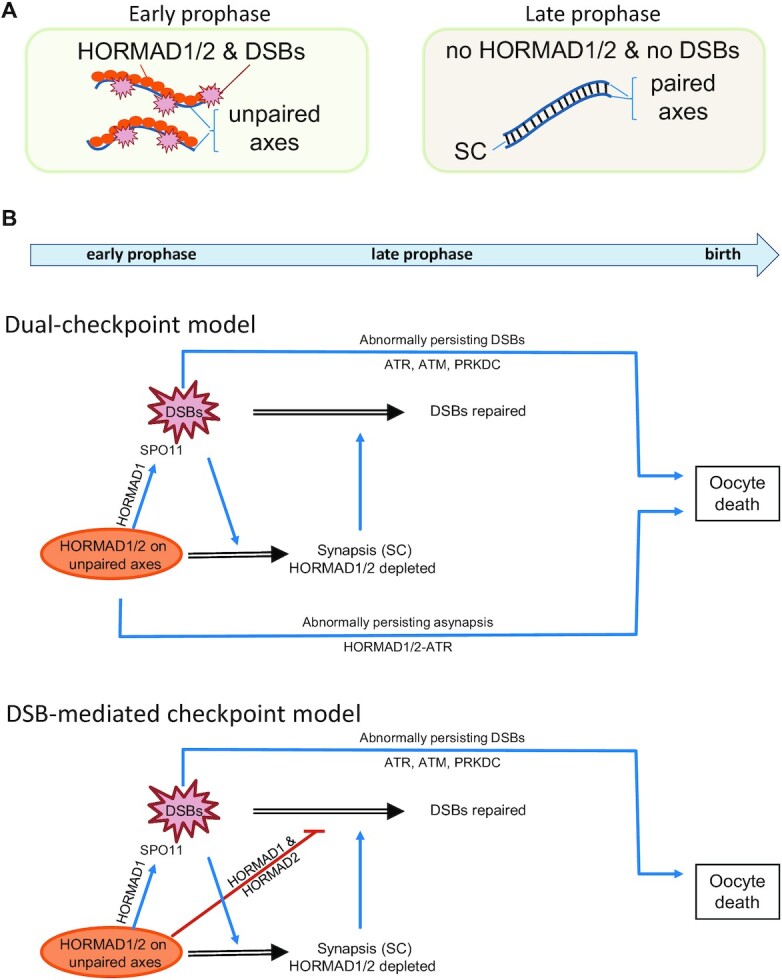
Models of prophase checkpoint in oocytes. (**A**) Schematics of chromosome configurations in early (left) and late (right) stages of meiotic recombination in normal meiocytes. (**B**) Models of prophase checkpoint in oocytes. Upper panel, dual checkpoint model: Persistent DSBs activate DNA damage sensor kinases, which leads to perinatal oocyte elimination if DSBs are unrepaired till late prophase (top checkpoint pathway). HORMAD1/2-dependent recruitment of ATR to unsynapsed axes activates an ATR signalling pathway (bottom pathway) that does not require DSBs. This pathway serves as a synapsis checkpoint mechanism that eliminates asynaptic oocytes perinatally. Lower panel, DSB-dependent checkpoint model: HORMAD1/2 activates the prophase checkpoint primarily by delaying DSB repair, which increases the steady state numbers of unrepaired DSBs. HORMAD1/2-dependent axis binding of ATR plays lesser or no direct role in checkpoint activation (hence, it is omitted from the scheme). Note that a combination of the two models is also possible. HORMAD1 has a role in enabling SPO11-mediated DSB formation in early prophase but not in late prophase. Hence, this function does not directly contribute to checkpoint activation in late prophase, but it is important for synapsis formation. Processes, activation and inhibition are marked by black double-line, blue and red arrows, respectively.

Meiotic DSB formation and repair are regulated by two meiotic chromosome structures, the chromosome axis and the synaptonemal complex (SC) [reviewed in (6–8), Figure 1]. The chromosome axis is a rod-like structure that is assembled by the oligomerization of two structural proteins, SYCP2 and SYCP3, on the longitudinal cohesion core of each sister chromatid pair after pre-meiotic DNA replication (9–11). Once chromosomes find their homologs, a zipper-like chromatin structure, the SC, forms in paired sections of homolog axes. The SC is a tripartite structure where aligned homolog axes are connected to a longitudinal central element by proteinaceous transverse filaments (9).

DSBs are formed on axes in large numbers, 200–400 in mice, by SPO11 (1,5). In early prophase, meiocytes avoid repairing these DSBs by non-homologous end joining (NHEJ) or recombination that uses sister chromatids as repair templates (inter-sister recombination), because neither of these DSB repair pathways promotes homolog pairing and resultant synapsis (6,7,12). Instead, meiocytes utilize inter-homolog recombination, where homologs serve as templates for DSB repair. Single-stranded DNA (ssDNA) ends that are generated from DSBs invade homologs with the help of two ssDNA-binding recombinases, DMC1, which has a catalytic role, and RAD51, which supports DMC1 (6,7,13,14). DNA strand invasions promote juxtaposition of homolog axes culminating in SC formation between each homolog pair by the pachytene stage of meiosis.

The SC is thought to effect feedback control on recombination in response to successful homolog pairing (6,8,15–17). In mice, unsynapsed axes provide permissive environment for DSB formation (15,17–21), thereby enabling DNA strand invasions and homology search. In contrast, unsynapsed axes do not support timely completion of DSB repair as turnover of early recombination markers is delayed in SC-defective mice (22–26). These axis functions are thought to involve two meiosis-specific proteins, HORMAD1 and HORMAD2, which preferentially bind to unsynapsed axes [(17,21,27–29) and Figure 1]. The SC is hypothesized to promote HORMAD1/2 depletion from axis (17,21), terminate DSB formation (8,15–17,21,30), and promote post-strand-invasion steps in recombination (6,7,17). In the latter role, synapsis jointly acts with the MSH4/MSH5 complex (MutSγ), which stabilizes DNA strand invasions thereby promoting the completion of DSB repair and crossover formation (6,31,32). By the end of prophase, the majority of DSBs are repaired by non-reciprocal inter-homolog recombination, manifesting as gene conversions, and at least one DSB is turned into crossover in each synapsed chromosome (6,7). Further, suppression of inter-sister recombination and NHEJ are thought to be lifted in and beyond late pachytene, to enable the repair of DSBs that are recalcitrant to synapsis-promoted inter-homolog recombination (7,12,33).

Genome integrity is safeguarded by a meiosis-specific prophase checkpoint that responds to DSB repair defects and synapsis failure (hereafter referred as asynapsis). In females, the prophase checkpoint eliminates oocytes around birth, where most wild-type oocytes dismantle SCs and chromosome axes in the diplotene and the ensuing dictyate stages of oogenesis (34). The prophase checkpoint involves HORMAD1 and HORMAD2, which serve as asynapsis sensors by preferentially binding to unsynapsed axes (17,21,35–38) (Figure 1A). Limited and pervasive asynapsis have been hypothesized to trigger oocyte elimination by distinct pathways (28,33,39,40). Limited asynapsis (up to 3 chromosome pairs) permits HORMAD1 and HORMAD2 to concentrate high levels of a DNA damage response (DDR) kinase, ATR, to unsynapsed chromatin, which leads to meiotic silencing of unsynapsed chromosomes (MSUC) (33,40,41). It is thought that MSUC-mediated silencing of *ad hoc* sets of essential genes underlie elimination of oocytes if asynapsis affects few chromosomes (33).

If asynapsis is extensive, MSUC is inefficient due to limiting amounts of the MSUC machinery (40,42), hence MSUC-independent checkpoint pathways are expected to act. Prior studies suggested two key alternative models (Figure 1B) that differ in the hypothesized functions of HORMAD1/2 regarding checkpoint activation in pervasively asynaptic oocytes (28,35). Hereafter, we refer to these alternatives as the dual-checkpoint and the DSB-dependent checkpoint models.

According to the dual-checkpoint model, asynaptic axes and DSB repair defects contribute to oocyte elimination via distinct pathways (Figure 1B upper panel). The synapsis-branch of the checkpoint depends on axis-bound HORMAD1/2, which recruit and activate ATR independent of DSBs on asynaptic chromosomes (21,35–37,43). Axis-associated ATR activation triggers perinatal oocyte apoptosis in persistently asynaptic oocytes (21,33,35–37). The DSB-branch of the dual-checkpoint promotes oocyte apoptosis by DDR signalling from abnormally persisting ssDNAs that originate from DSBs (34,44–46).

According to the DSB-dependent checkpoint model (Figure 1B, lower panel), pervasive asynapsis leads to checkpoint activation because axis-bound HORMAD1/2 hinder DSB repair, thereby increasing the numbers of unrepaired ssDNAs and resultant DDR signalling (27,28). Thus, ATR signalling from asynaptic axes plays minor or no role, instead perinatal oocyte elimination primarily relies on DDR signalling from persistent DSBs (28,39).

*Spo11^–^^/^^–^* oocytes are devoid of programmed DSBs (1,5), are asynaptic, and are eliminated around birth (34) in a HORMAD1/2-dependent manner (21,35–37). These observations initially gave rise to the idea of a DSB-independent synapsis checkpoint consistent with the dual-checkpoint model. However, curiously, SPO11-deficient oocytes accumulate RAD51 foci indicative of unrepaired DSBs of unknown origin (47). Further, RAD51 foci depend on HORMAD2 in *Spo11^–^^/^^–^* oocytes – dependence on HORMAD1 was not tested (28). Therefore, it was proposed that HORMAD1/2 prevented timely DSB repair in asynaptic chromosomes, leading to the persistence of DSBs above a threshold (≥10 DSBs in mice) that effectively induced perinatal apoptosis in *Spo11^–^^/^^–^* oocytes consistent with the DSB-dependent checkpoint model (Figure 1B, lower panel) (28).

We tested the two alternative meiotic checkpoint models by analyzing DSB repair foci in two asynaptic mouse models, *Spo11^–^^/^^–^* and *Mcmdc2^–^^/^^–^*, where the latter, but not the former, are deficient in the meiosis-specific DSB repair machinery (48). We utilized diverse protein markers of ssDNAs and a method for direct ssDNA detection by BrdU–labelling. Surprisingly, our data indicate that RAD51 foci do not represent ssDNAs in *Spo11^–^^/^^–^* oocytes. This observation questions if oocytes are eliminated due to DDR signalling from ssDNAs in *Spo11^–^^/^^–^* mice. We also found that despite persistence of HORMAD1/2 on asynaptic chromosome axes, most ssDNAs and corresponding DSB repair foci disappeared by birth in *Mcmdc2^–^^/^^–^* oocytes. Thus, HORMAD1/2 does not efficiently block repair and/or turnover of ssDNAs in late meiotic prophase. Together, these observations provide strong evidence for a synapsis checkpoint that utilizes DDR signalling from asynaptic axes, and that does not require elevated numbers of persistent ssDNAs.

## MATERIALS AND METHODS

### Animal experiments

Gonads were collected from mice after euthanasia. Most cytological experiments of spermatocytes were carried out on samples collected from adult mice, unless indicated otherwise. *Mcmdc2* (48), *Spo11* (1), *Dmc1* (49), *Hormad1* (21) and *Hormad2* (35) mutant mice were used and maintained in accordance with the German Animal Welfare legislation (Tierschutzgesetz). The mice were kept in the barrier facility in individually ventilated cages at 22–24°C and 50–55% air humidity with 14-h light/10-h dark cycle. The feed was a rat–mouse standard diet in the form of pellets. The stocking density in the used cage type IIL was maximum five mice. Hygiene monitoring was carried out according to FELASA guidelines. All procedures pertaining to animal experiments were approved by the Governmental IACUC (Landesdirektion Sachsen) and overseen by the animal ethics committee of the Technische Universität Dresden. The license numbers concerned with the present experiments with animals are DD24-5131/287/1.

### Preparation of spermatocyte spreads

Preparation and immunostaining of nuclear surface spreads of spermatocytes was carried out according to earlier described protocols with minor modifications (18,50). Briefly, testis cell suspensions were prepared in PBS pH 7.4, then mixed with hypotonic extraction buffer in 1:1 ratio and incubated for 8 min at room temperature. After diluting the cell suspension five times in PBS pH 7.4, cell suspensions were centrifuged for 5 min at 1000 × g, and cells were resuspended in the 1:2 mixture of PBS and 100 mM sucrose solution. Cell suspensions were added to seven times higher volume (15 μl to 100 μl or 2–3 μl to 15 μl droplets) of filtered (0.2 μm) 1% paraformaldehyde (PFA), 0.15% Triton X-100, 1 mM sodium borate pH 9.2 solution on diagnostic slides, and incubated for 60 min at room temperature in wet chambers. Nuclei were then dried for at least 1 h under fume-hood. Finally, the slides were washed in 0.4% Photo-Flo 200 (Kodak, MFR # 1464510), rinsed with distilled water and dried at room temperature.

### Preparation of oocyte spreads

To prepare nuclear surface spread oocytes, two ovaries from each mouse were incubated in 20 μl hypotonic extraction buffer for 15 min (Hypotonic Extraction Buffer/HEB: 30 mM Tris–HCl, 17 mM trisodium citrate dihydrate, 5 mM EDTA, 50 mM sucrose, 0.5 mM DTT, 0.5 mM PMSF, 1× Protease Inhibitor Cocktail). After incubation, HEB solution was removed and 16 μl of 100 mM sucrose in 5 mM sodium borate buffer (pH 8.5) was added. Ovaries were punctured by two needles to release oocytes. Big pieces of tissue were removed. 9 μl of 65 mM sucrose in 5 mM sodium borate buffer (pH 8.5) was added to the cell suspension and incubated for 3 min. After mixing, 1.5 μl of the cell suspension was added in a well containing 20 μl of fixative (1% paraformaldehyde, 50 mM borate buffer pH 9.2, 0.15% Triton X-100) on a glass slide. Cells were fixed for 45 min in humid chambers, then slides were air dried on bench. Upon completion of drying, slides were washed with 0.4% Photo-Flo 200 solution (Kodak, MFR # 1464510) for 5 min, and afterwards, they were rinsed with distilled water and further air dried at room temperature.

### Immunofluorescence on gonad sections

To detect apoptosis in ovary sections, we sectioned ovaries after fixation. Ovaries from newborn mice were fixed in 3.6% formaldehyde in PBS pH 7.4, 0.1% Triton X-100 at room temperature for 20 min. After fixation, ovaries were washed 3 times in PBS pH 7.4 and placed in 30% sucrose overnight at 4°C. Fixed ovaries were frozen on dry ice in O.C.T. compound (Sakura Finetek Europe). 5 μm thick ovaries sections were cut and dried onto slides. Ovary sections were permeabilized by incubating the slides for 10 min in methanol and 1 min in acetone at –20°C. The sections were washed in PBS pH 7.4 and immediately used for immunofluorescence staining. Anti-cleaved-PARP (apoptosis marker) and GCNA1 (oocyte marker) (51) were detected on oocyte sections. The numbers of cleaved PARP-positive and -negative oocytes were counted on every seventh section to determine the proportion of apoptotic oocytes.

### Staining procedures

To immunostain spread nuclei and sections, slides were blocked with either 2.5% (w/v) BSA and 0.05% Tween in PBS pH 7.4 (most stainings) or with 1% Normal Goat Serum, 3% BSA, 0.02% Triton X-100, 0.02% NaN_3_ in TBS pH 7.6 (for staining of 15.5 dpc oocytes) for 1 h, then slides were incubated with primary antibodies diluted in blocking solution either for 3 h at room temperature or overnight at 4°C. Subsequently, slides were washed (3×) in PBS with 0.05% Triton X-100 (PBS-T) and incubated with secondary antibodies in blocking buffer at room temperature for 1 hour. Finally, slides were washed (3×) in PBS-T and embedded in SlowFade™ Gold Antifade Mountant with or without DAPI (Invitrogen).

### Labelling spermatocytes with BrdU

BrdU was administered to adult male mice in drinking water at 1 mg/ml concentration for 14 days. Drinking bottles containing BrdU solution were covered with aluminium foil to reduce exposure to light. The BrdU solution was refreshed every 3 days. Chromosome spreads were prepared as described above.

### Labelling oocytes with BrdU

Female mice were placed in a cage with a single male mouse overnight for 15.5 dpc embryos and for 3 days (to increase chances of mating) for newborns, after which females were moved into a different cage. Putative pregnancy was determined by weighing the female mice (52). Whereas the weights of most unfertilized females fluctuated less than ±1.5 g in the weeks following the separation from males, successfully fertilized female mice gained ∼1.75 g weight 7.5 days after fertilization. BrdU was administered to pregnant female mice in drinking water at 0.8 mg/ml concentration from 10 to 16 dpc for newborns. For the labelling of 15.5 dpc embryos, BrdU was administered until mice were euthanized at 15.5 dpc for oocyte spreads.

### Detection of ssDNA with BrdU in spermatocytes and oocytes

The nuclear spreads were treated with 50 μg/ml pepsin for 3 min at room temperature and then washed with PBS (3×). Further, spreads were treated with 0.05% trypsin and 0.01% CaCl_2_ for 10 min at 37°C and washed with PBS (3×). The spreads were blocked as described above and BrdU was visualized by indirect immunofluorescence staining. Meiocyte spreads were incubated in 1:50 dilution of mouse monoclonal anti-BrdU antibody (BD, Lot 7324574) in blocking buffer for 3 h at room temperature. Subsequently, slides were washed (3×) in PBS-T and incubated with secondary antibodies in blocking buffer at room temperature for 1 h. After 3 washes with PBS-T, the stained spreads were incubated with 4% PFA for 15 min and further washed with water and mounted with mounting media. All incubations were performed in closed wet chambers and for detection of BrdU foci in any experiment required this treatment. To test BrdU incorporation into nuclear DNA, the slides were denatured in 70% formamide/2× SSC for 10 min at 80°C for oocytes and 5 min for spermatocytes and then dehydrated in an alcohol series of ice cold ethanol; 70%, 85%, 100% for 2 min each. The slides were air dried and stained for BrdU and SYCP3.

### Quantification of DSB repair foci/ssDNAs

**Figure 2.**
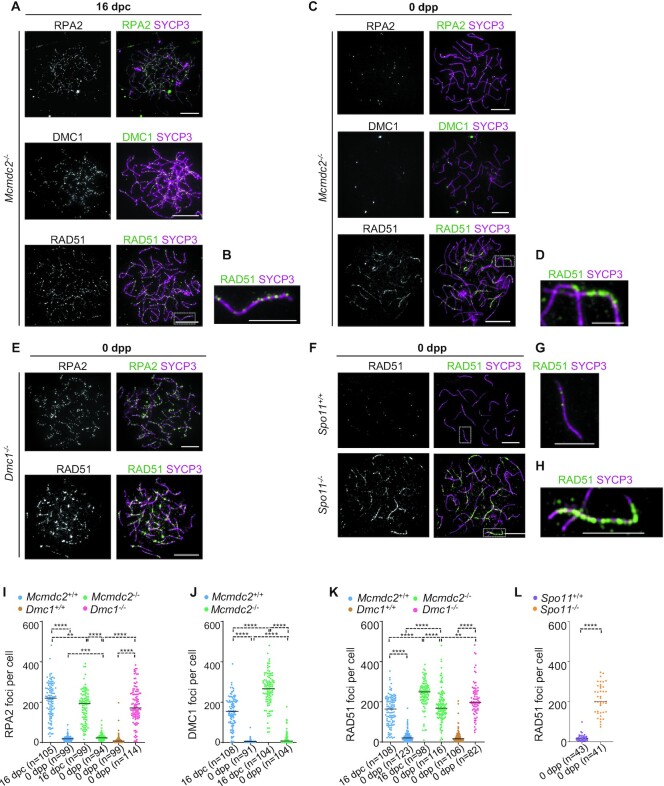
ssDNA repair intermediates turnover in *Mcmdc2^–/–^* oocytes. (**A–H**) Chromosome axis (SYCP3) and recombination proteins RPA2, DMC1 or RAD51 were detected by immunofluorescence in surface-spread oocytes of (**A**, **B**) 16 dpc fetuses and (**C**–**H**) newborn mice (0 dpp) of the indicated genotypes. (**B**, **D**, **G**, **H**) Enlarged insets of RAD51 foci in oocytes of (**B**) 16 dpc *Mcmdc2^–/–^* fetuses and newborn (**D**) *Mcmdc2^–/–^*, (**G**) *Spo11^+/+^* or (**H**) *Spo11^–/–^* mice are shown. Bars, 10 μm; in enlarged insets, 5 μm. (**I**–**L**) Quantification of axis-associated (**I**) RPA2, (**J**) DMC1 and (**K, L**) RAD51 focus numbers in the indicated genotypes at 16 dpc and 0 dpp time points. *n* = numbers of analysed cells from at least two animals. Bars mark medians, (**I**) 220 and 18 in *Mcmdc2^+/+^* oocytes at 16 dpc and 0 dpp, 192 and 22.5 in *Mcmdc2^–/–^* oocytes at 16 dpc and 0 dpp, 7 and 171 in *Dmc1^+/+^*and *Dmc1^–/–^* oocytes at 0 dpp, (**J**) 154 and 4 in *Mcmdc2^+/+^* oocytes at 16 dpc and 0 dpp, 266.5 and 7 in *Mcmdc2^–/-^* oocytes at 16 dpc and 0 dpp, (**K**) 164 and 19 in *Mcmdc2^+/+^* oocytes at 16 dpc and 0 dpp, 249 and 168.5 in *Mcmdc2^–/–^* oocytes at 16 dpc and 0 dpp, 16 and 198 in *Dmc1^+/+^*and *Dmc1^–/–^* oocytes at 0dpp, (**L**) 16 and 201 in *Spo11^+/+^*and *Spo11^–/–^* oocytes at 0 dpp, respectively. Mann–Whitney *U* test, 0.001 < *P*< 0.01 (**), 0.0001 < *P*< 0.001 (***), and *P*< 0.0001 (****). See also Supplementary Figure S1.

We manually counted recombination-protein and ssDNA foci. Recombination foci closely associate with chromosome axes throughout meiosis. Therefore, in the case of RPA2 (Figures 2, 5 and 7), RAD51, DMC1 and BrdU we considered only foci that overlapped or closely associated with chromosome axes. This method was used to minimize the effect of punctate aspecific staining, which occasionally was present in nuclear spreads off axis. In the case of RPA2 in Figure 6C, D and Supplementary Figure S5A an alternative method was used to distinguish specific from aspecific staining. Antibodies that were raised against RPA2 in rabbit and rat were used to co-stain meiocytes. We considered foci specific only if they were detected by both antibodies; in this method, the association between foci and axes was not evaluated.

### Statistical analysis

Statistical analysis of cytological observations was done by GrapPad Prism 7 and lme4 programming package in R (likelihood ratio test). All tests and *P* values are provided in the corresponding legends and/or figures.

### Biological materials availability

Transgenic mouse strains, analysis scripts and pipelines used in this study are available from the authors upon request.

## RESULTS

### Differential focus formation of single-stranded DNA markers in MCMDC2-deficient oocytes

Our previous work identified a meiosis-specific protein, MCMDC2, that enables the accumulation of MSH4/MSH5 (MutSɣ) complex in recombination foci (48). Accordingly, MCMDC2 was hypothesized to promote the stabilization of DNA strand invasions during homology search. Consistent with this hypothesis, MCMDC2-deficiency resulted in extensive asynapsis, a lack of crossover-specific recombination foci, and an abnormal persistence of early recombination markers in meiocytes of both sexes. Thus, *Mcmdc2^–^^/^^–^* oocytes had abnormally high numbers of RAD51 and DMC1 foci at 18.5 days post coitum (dpc), where most oocytes reach late pachytene in wild type. These defects were coupled with loss of oocytes by adulthood (48). Together, these observations suggested that *Mcmdc2^–^^/–^* oocytes are eliminated by a DSB-dependent checkpoint mechanism at or soon after birth. To further test this hypothesis we examined if unrepaired DSB markers persist beyond 18.5 dpc, until 0 days postpartum (dpp), where unrepaired DSBs are thought to trigger apoptosis in most oocytes (53).

Therefore, we compared focus numbers of ssDNA-binding proteins, RAD51, DMC1 and RPA2, in oocytes at 16 dpc and 0 dpp, where late zygotene and early-mid pachytene (16 dpc) or diplotene (0 dpp) stages were prevalent. We note that meiotic recombination defects do not alter significantly the developmental timing of chromosome axis formation and disassembly. Therefore, matching developmental time points are thought to allow comparison of equivalent prophase stages in wild type and recombination mutants. All three recombination markers formed high numbers of foci in both wild-type and *Mcmdc2^–^^/–^* oocytes at 16 dpc (Figure 2 and Supplementary Figure S1). DSB repair foci diminished in wild-type oocytes by 0 dpp indicating the repair of most DSBs by diplotene (Figure 2I-L and Supplementary Figure S1). Despite extensive asynapsis both DMC1 and RPA2 focus numbers were much lower (38- and 8.5-fold, respectively) in *Mcmdc2^–^^/-^* oocytes at 0 dpp as compared to 16 dpc (Figure 2I, J). In contrast, RAD51 focus numbers remained high in *Mcmdc2^–^^/–^* oocytes at 0 dpp (Figure 2K). These observations suggest that the nature of recombination intermediates and/or the recombination machinery significantly changes in *Mcmdc2^–^^/–^* oocytes as prophase progresses. To test if preferential persistence of RAD51, in comparison with other recombination markers, is a general feature of recombination-defective oocytes where ssDNA-rich intermediates persist, we detected RPA2 and RAD51 in *Dmc1^–^^/–^* oocytes. DMC1 is the main recombinase that catalyzes DNA strand invasions in meiosis, hence absence of DMC1 leads to the accumulation of unrepaired ssDNA ends at DSB sites (13,54). Both RAD51 and RPA2 foci were present in high numbers in *Dmc1^–^^/-^* oocytes at 0 dpp contrasting *Mcmdc2^–^^/–^* oocytes (Figure 2E, I, K). Thus, RPA2 foci are depleted from chromosomes in the absence of MCMDC2 but not in the absence of DMC1. These observations suggest that the fates of recombination intermediates significantly differ in *Mcmdc2^–^^/^^–^* and *Dmc1^–^^/–^* oocytes, despite a severe failure in inter-homolog recombination in both genotypes.

In contrast to focal RAD51 staining patterns, which were observed in 16 dpc oocytes, RAD51 appeared to accumulate in densely packed foci and/or axially-elongated filaments in *Mcmdc2^–^^/–^* oocytes at 0 dpp (Figure 2B, D). Similar filamentous RAD51 accumulations were reported on chromosomes also in *Spo11^–^^/^^–^* oocytes (at 17.5 dpc (47) and 0 dpp (28) Figure 2F–H, L), where programmed DSBs do not form. The filamentous RAD51 complexes were postulated to mark ssDNAs resulting from enigmatic SPO11-independent DSBs in the *Spo11^–^^/–^* genotype, but prior reports differed on whether or not another ssDNA-binding protein, RPA, forms high numbers of foci in SPO11-deficient oocytes (47,55). It is uncertain if filamentous RAD51 foci have similar origin in *Spo11^–^^/^^–^* and *Mcmdc2^–^^/^^–^* oocytes. Nonetheless, the unusual behavior of RAD51 foci and the depletion of both DMC1 and RPA2 foci in oocytes of newborn *Mcmdc2^–^^/^^–^* mice raise the question if filamentous RAD51 complexes represent *bona fide* ssDNA-associated recombination intermediates in *Spo11^–^^/^^–^* and *Mcmdc2^–^^/^^–^* oocytes at 0 dpp.

### Direct detection of ssDNA by BrdU staining

**Figure 3.**
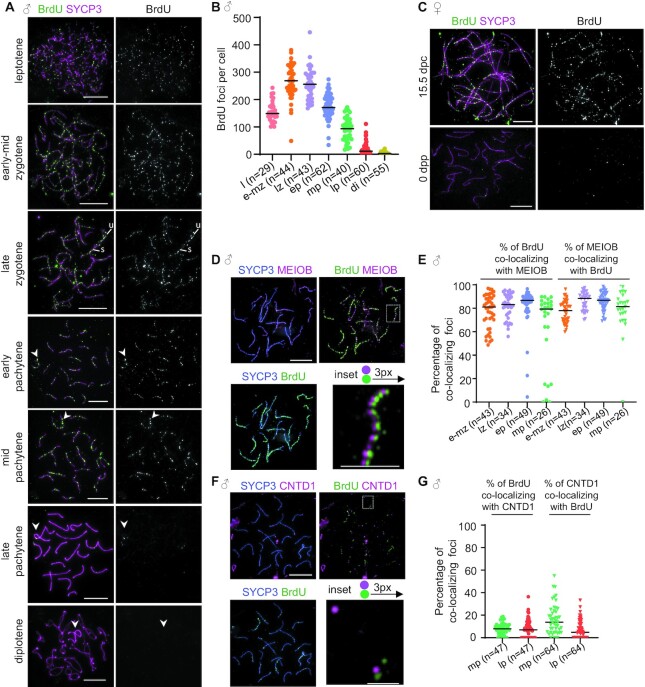
Direct detection of ssDNAs by BrdU labeling. (**A, C, D, F**) Immunofluorescence staining of indicated proteins and BrdU in BrdU-labelled wild-type surface-spread (**A, D, F**) spermatocytes from adult mice or (**C**) oocytes from 15.5 dpc fetus or 0 dpp mice. (**D, F**) BrdU signal is shifted to the right with three pixels in the enlarged insets to facilitate detection of overlapping signals. Unsynapsed (u) and synapsed (s) regions are marked. Arrowhead mark sex chromosomes Bars, 10 μm; in enlarged insets, (**D**) 5 μm and (**F**) 2 μm. (**B, E, G**) Quantifications of (B) BrdU foci or the colocalization of BrdU foci with (E) MEIOB or (G) CNTD1 in spermatocytes. *n* = number of spermatocytes from two analysed mice. Analysed stages are indicated: leptotene (l), early-mid zygotene (e-mz), late zygotene (lz), early pachytene (ep), mid pachytene (mp), late pachytene (lp), and diplotene (di). Bars mark medians, (B) 149 (l), 269 (e-mz), 256 (lz),171 (ep), 93.5 (mp), 11.5 (lp) and 3 (di), (E) % of BrdU: 81 (e-mz), 83 (lz), 86.7 (ep), 79.3 (mp), % of MEIOB: 77.9 (e-mz), 88.4 (lz), 86.8 (ep), 81.4 (mp), (G) % of BrdU: 7.9 (mp), 6.9 (lp), % of CNTD1: 13.8 (mp), 4.8 (lp).

To answer if ssDNAs are present in *Mcmdc2^–^^/^^–^* oocytes we sought a method that allows direct detection of ssDNA as opposed to the detection of ssDNA-binding proteins. A monoclonal anti-bromodeoxyuridine (BrdU) antibody detects BrdU labelling only in ssDNA but not double-stranded DNA (56), due to the inaccessibility of the epitope in the latter (Supplementary Figure S2A, B). This observation was utilized previously to detect DNA lesions that associate with ssDNAs both in somatic cells and spermatocytes (56). Surprisingly, BrdU foci were detected in much lower numbers (20–30) than ssDNA-binding proteins (in excess of 200) in spermatocytes cells (56), which raised the possibility that BrdU may be masked by recombination proteins in ssDNAs in meiocytes. To achieve efficient detection of ssDNA, we exposed nuclear spread spermatocytes and oocytes to controlled digestion by trypsin and pepsin before anti-BrdU staining. This approach enabled detection of BrdU foci in numbers that matched predicted DSB numbers in meiocytes (Figure 3).

BrdU labelling did not cause obvious defects in homolog pairing and synapsis, suggesting that meiotic recombination was not significantly affected. BrdU foci were mainly detected on chromosome axes consistent with the reported association of recombination intermediates with axes (Figure 3A, C, D, F and Supplementary Figure S2B). BrdU focus numbers peaked in early-mid zygotene (median 269, mean 265, *n* = 44) and gradually declined upon progression to late pachytene and beyond as showed by quantification in spermatocytes (Figure 3B). Consistently, BrdU foci were abundant in oocytes at 15.5/16 dpc (median 168.5 and mean 172.4, *n* = 37 oocytes), where late zygotene and early pachytene stages dominate, but BrdU foci diminished (median 5, mean 7.68, *n* = 151 oocytes) as oocytes progressed to late pachytene and diplotene in newborn mice (Figure 3C). Due to loss of antibody reactivity following trypsin and pepsin treatment, we could not examine co-localization between BrdU staining and most of the known recombination proteins, including RAD51, DMC1, RPA1/2, MLH1 or PRR19 (Supplementary Table S1). Nonetheless, we found a very high degree of co-localization between foci of BrdU and MEIOB (median 83–88% in late zygotene spermatocytes, Figure 3D, E). MEIOB is a meiosis-specific component of an RPA protein complex, which is thought to mark ssDNAs in recombination intermediates during meiotic recombination (Supplementary Figure S2C) (57,58). Hence, we conclude that BrdU staining efficiently detects recombination intermediates that contain ssDNA in meiosis. In contrast to extensive co-localization between BrdU and MEIOB, the few BrdU foci that remained in mid and late pachytene rarely co-localized (7 and 8%, respectively) with crossover-specific recombination complexes, which were detected by CNTD1 staining (Figure 3F, G) (59). Crossover-specific recombination complexes are thought to associate with double Holliday junctions, which contain very little ssDNA (Supplementary Figure S2C). Hence, infrequent BrdU-CNTD1 co-localization reconfirms that BrdU staining is restricted to recombination intermediates that contain considerable ssDNA tracks. Thus, BrdU focus kinetics match the prevailing model of meiotic recombination (Supplementary Figure S2C), according to which (i) ssDNAs are abundant during leptotene to zygotene stages, where most DNA strand exchange is initiated, but (ii) ssDNAs are diminished as DNA strand exchange intermediates are resolved when meiocytes progress to and beyond pachytene.

### ssDNAs are rare in MCMDC2-deficient oocytes in newborn mice

**Figure 4.**
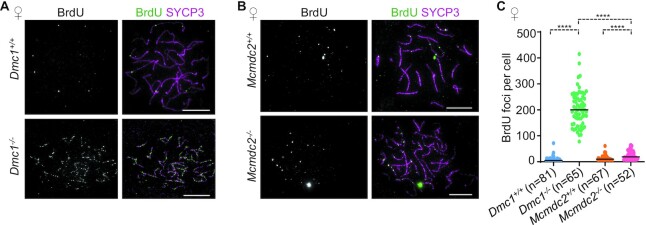
ssDNAs diminish in asynaptic *Mcmdc2^–/–^* oocytes in late prophase. (A, B) Chromosome axis (SYCP3) and BrdU were detected by immunofluorescence in surface-spread oocytes of newborn (0 dpp) mice. Images show littermate pairs of (**A**) *Dmc1^+/+^* and *Dmc1^–/–^* or (**B**) *Mcmdc2^+/+^*and Mcmdc2*^–/–^* mice. Bars, 10 μm. (**C**) Quantification of axis-associated BrdU focus numbers in mixtures of late pachytene and early diplotene oocytes in 0 dpp mice in the indicated genotypes. *n* = numbers of analysed cells from two mice; medians (bars) are 5 in *Dmc1^+/+^*, 200 in *Dmc1^–/–^*, 10 in *Mcmdc2^+/+^*and 19 in *Mcmdc2^–/–^* oocytes. Mann–Whitney *U* test, *P*< 0.0001 (****).

We employed BrdU labelling to test if recombination intermediates that contain extensive ssDNAs persist in *Mcmdc2^–^^/^^–^* oocytes at 0 dpp. We focused on the analysis of oocytes where chromosome axes were fully formed, indicating a prophase stage that was equivalent to late pachytene and early diplotene in wild type. *Mcmdc2^–^^/–^* oocytes had slightly more BrdU foci (median, 19) than wild-type oocytes (median, 5–10) but much less than *Dmc1^–^^/^^–^* oocytes (median, 200) (Figure 4). BrdU focus numbers matched RPA2 but not RAD51 focus numbers in oocytes of newborn mice of the examined genotypes (compare Figures 2 and 4). These observations strongly suggest that, contrary to expectations, most of the intense filamentous RAD51 staining (Figure 2C, D) does not represent extensive ssDNAs in *Mcmdc2^–^^/^^–^* oocytes at 0 dpp. Therefore, we do not consider axis-associated RAD51-labelling as a reliable marker of ssDNAs in late prophase stages. In contrast, RPA2 and DMC1 foci seem to reliably reflect the presence of ssDNAs. Together, BrdU and RPA2 stainings show that most DSBs are not repaired, and that resected ssDNA ends persist until late prophase in the absence of DMC1. In contrast, ssDNAs diminish in both wild-type and *Mcmdc2^–^^/^^–^* oocytes by 0 dpp. This observation suggests that despite defective synapsis and defective homolog pairing most recombination intermediates are repaired or turned into advanced recombination intermediates without extensive ssDNAs (hereafter, repair/turnover of ssDNAs) in *Mcmdc2^–^^/^^–^* oocytes (Figures 2 and 4, (48)). It follows that persistent RAD51 accumulations mark either undamaged double-stranded DNA (dsDNA) or recombination intermediates that contain little ssDNAs in *Mcmdc2^–^^/^^–^* oocytes. Persistent RAD51 accumulations that do not represent ssDNAs did not seem to cause prophase arrest by DDR in somatic cell models where RAD51 was overexpressed and/or RAD54 family translocases were depleted (60). Instead, ssDNA-independent RAD51 caused cell toxicity by destabilizing the genome during chromosome segregation. Therefore we disfavor the idea that ssDNA-independent RAD51 accumulations significantly contribute to the elimination of prophase stage oocytes in *Mcmdc2^–^^/^^–^* mice. In contrast, we note that most *Mcmdc2^–^^/^^–^* oocytes (42 out of 52, or 81%) had BrdU foci in numbers that equaled or exceeded the 10-DSB-threshold that is thought to trigger apoptosis in oocytes perinatally (28). Therefore, despite the repair or processing of most ssDNAs, persistent ssDNAs likely make a major contribution to oocyte apoptosis in *Mcmdc2^–^^/^^–^* mice.

Defective recombination is associated with elimination of both *Dmc1^–^^/^^–^* and *Mcmdc2^–^^/^^–^* oocytes before adulthood (34,48). Hence, we considered the possibility that repair/turnover of ssDNAs occurs only in a small subset of *Mcmdc2^–^^/^^–^* oocytes, but preferential elimination of oocytes that have high numbers of ssDNAs may result in low DSB repair focus numbers in the surviving pool of *Mcmdc2^–^^/^^–^* oocytes at 0 dpp. However, oocyte numbers were similar in ovaries of wild type and *Mcmdc2^–^^/^^–^* mice at 0 dpp (Supplementary Figure S3A, B), arguing against the idea that excess apoptosis resulted in low DSB focus counts in *Mcmdc2^–^^/^^–^*. Further, oocyte numbers were lower, and rates of apoptosis were higher in ovaries of *Dmc1^–^^/^^–^* as compared to *Mcmdc2^–^^/^^–^*mice (Supplementary Figure S3), indicating that a larger number of unrepaired ssDNAs constitute a stronger DNA damage signal that triggers apoptosis earlier in *Dmc1^–^^/^^–^* as compared to *Mcmdc2^–^^/^^–^* oocytes. It also follows that early apoptosis of the most defective oocytes cannot explain lower DSB-repair foci numbers in *Mcmdc2^–^^/^^–^*as compared to *Dmc1^–^^/^^–^* oocytes. These observations support the conclusion that repair and/or turnover of ssDNAs are primarily responsible for a depletion of ssDNA-containing recombination foci in *Mcmdc2^–^^/^^–^* oocytes by 0 dpp.

### DMC1 is required for the depletion of ssDNA foci in *Mcmdc2^–/–^* oocytes

**Figure 5.**
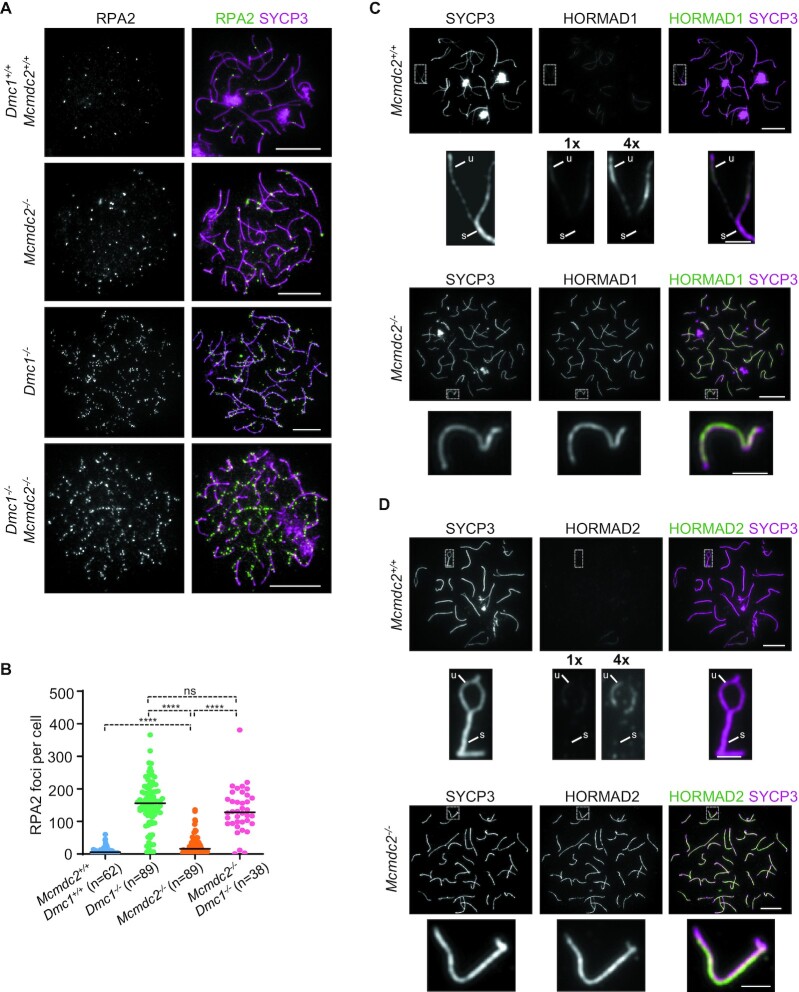
Turnover of RPA2 foci requires DMC1 in *Mcmdc2^–/–^* oocytes. (A, C, D) Chromosome axis (SYCP3) and (**A**) RPA, (**C**) HORMAD1 or (**D**) HORMAD2 were detected by immunofluorescence in surface-spread oocytes of newborn (0 dpp) mice in the indicated genotypes. (C, D) Enlarged insets show desynapsing and asynaptic chromosomes in *Mcmdc2^+/+^* and *Mcmdc2^–/-^* oocytes, respectively. Unsynapsed (u) and synapsed (s) regions are marked. (C) HORMAD1 and (D) HORMAD2 signals are equivalently leveled (1×) or four times amplified (4×) in the images of *Mcmdc2^+/+^* oocytes as compared to *Mcmdc2^–/–^* oocytes, to illustrate higher HORMAD1/2 accumulation in *Mcmdc2^–/–^* oocytes. Bars, 10 μm; in enlarged insets, 2 μm. (**B**) Quantifications of axis-associated foci of RPA2 in oocytes of newborn mice in the indicated genotypes; medians (bars) are 5.5 in *Dmc1^+/+^ Mcmdc2^+/+^*, 156 in *Dmc1^–/–^*, 16 in *Mcmdc2^–/–^* and 128 in *Mcmdc2^–/–^ Dmc1^–/–^*oocytes. *n* = numbers of analysed cells from two animals. Mann–Whitney *U* test, non-significant *P* > 0.05 (ns), *P*< 0.0001 (****).

Timely repair of meiotic DSBs requires inter-homolog DNA strand invasions and SC formation, both of which are enabled by the meiosis-specific recombinase, DMC1 (13,14). Accordingly, DMC1-defficiency causes asynapsis and an inability to repair DSBs leading to perinatal oocyte elimination (34,49,61). Curiously, the repair/turnover of ssDNAs do not require synapsis or homolog alignment in *Mcmdc2^–^^/^^–^* oocytes as evidenced by diminished ssDNA-containing recombination foci despite pervasive homolog pairing/synapsis failure (Figures 2 and 4, (48)). It follows that *Mcmdc2^–^^/^^–^* oocytes may utilize DMC1-independent repair pathways that do not require synapsis or inter-homolog strand invasions; relevant pathways may involve NHEJ and inter-sister recombination, which are normally suppressed in wild-type meiosis. To test this hypothesis we detected DSB repair foci in oocytes of newborn *Mcmdc2^–^^/^^–^ Dmc1^–^^/–^* mice (Figure 5A). High numbers of RPA2 foci were present in both *Dmc1^–^^/^^–^* and *Mcmdc2^–^^/^^–^ Dmc1^–^^/^^–^* oocytes (Figure 5A, B), which contrasted with *Mcmdc2^–^^/^^–^* oocytes, where RPA2 focus numbers were low (Figure 5A, B). We conclude that DMC1 and/or DMC1-mediated DNA strand invasions into homologs or sister chromatids are necessary for synapsis-independent repair/turnover of ssDNAs in *Mcmdc2^–^^/^^–^* oocytes in late prophase.

### HORMAD1 and HORMAD2 are present on unsynapsed axes in *Mcmdc2^–/–^* oocytes

Asynapsis is thought to result in delayed repair of DSBs as evidenced by persistence of ssDNA-containing recombination foci in SC-defective meiocytes (22–26). The barrier to efficient DSB repair on unsynapsed axes was hypothesized to involve HORMAD1 and HORMAD2 (17,27–29). It has been speculated that HORMAD1 and HORMAD2 block or slow down all types of DSB repair (17,29), or that HORMADs selectively block NHEJ or inter-sister recombination (27–29). Given that despite prevalent asynapsis ssDNA foci diminish in *Mcmdc2^–^^/^^–^* oocytes (Figure 4) we tested if MCMDC2 was required for HORMAD1 and HORMAD2 presence on unsynapsed axes. Only low levels of HORMAD1 and HORMAD2 associated with desynapsing chromosome axes in early diplotene oocytes of wild-type newborn mice (Figure 5C, D). In contrast, high levels of HORMAD1 and HORMAD2 were present on chromosome axes in all *Mcmdc2^–^^/^^–^* oocytes (*n* > 100) that had fully developed axes, which is characteristic of late pachytene and early diplotene at 0 dpp (Figure 5C, D). Thus, ssDNAs diminish (Figures 2 and 4) even in the presence of high levels of axis-bound HORMAD1 and HORMAD2 in *Mcmdc2^–^^/^^–^* oocytes. Interestingly, low recombination foci numbers were also reported in asynaptic perinatal oocytes in MCMDC2-proficient backgrounds (22,33). Together, these observations suggest that axial HORMAD1 and HORMAD2 do not efficiently block DSB repair on unsynapsed chromosomes in perinatal oocytes of MCMDC2-proficient or -deficient mice.

### ssDNA focus numbers are lower in *Spo11^–/–^* than wild type oocytes

The DSB-dependent oocyte checkpoint model (Figure 1B lower panel) emerged from the observation that SPO11-deficient oocytes accumulate high numbers of RAD51 foci (occasionally in excess of 100) (47) on a HORMAD2-dependent manner (28) soon before or around birth. We observed that HORMAD1 was also required for the accumulation of RAD51 foci in *Spo11^–^^/^^–^* oocytes (Supplementary Figure S4). However, our data suggest that RAD51 is not a reliable marker of ssDNAs in perinatal oocytes (see above and Figures 2 and 4).

**Figure 6.**
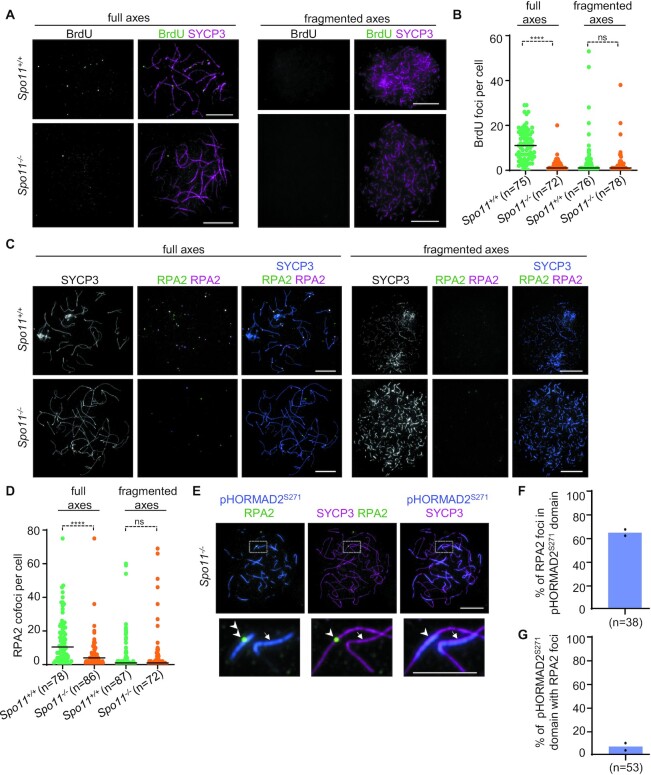
*Spo11^–/–^* oocytes contain less or equivalent numbers of ssDNA foci as compared to wild-type oocytes. (A, C, E) Chromosome axis (SYCP3), (E) pHORMAD2^S271^ and (**A**) BrdU or (**C, E**) RPA2 were detected by immunofluorescence in surface-spread oocytes of newborn (0 dpp) *Spo11^+/+^* and *Spo11^–/–^* mice. (A) and (C) show oocytes whose axes are fully formed (corresponding to late pachytene and early diplotene) or fragmented (corresponding to late diplotene). (C) RPA2 was detected by antibodies from both rat and rabbit to allow high specificity detection of RPA2 foci. (E) Enlarged insets show asynaptic axes whose segments acquired pHORMAD2^S271^ either in the presence or absence or RPA2 focus. (A, C, E) Bars, 10 μm; in enlarged insets, 5 μm. (**B**, **D**) Quantification of axis-associated (B) BrdU foci or (D) RPA2 cofoci, i.e. foci simultaneously detected by rat and rabbit anti-RPA2 antibodies, in the oocytes of 0 dpp mice of the indicated genotypes in either (B) CD-1 or (D) C57BL/6J backgrounds. Focus counts are shown in oocytes where axis is either fully formed or fragmented. Medians (bars) are 11, 1, 1 and 1 in (B) and 10.5, 4, 1 and 1 in (D) from left to right, respectively. Mann–Whitney *U* test, non-significant *P* > 0.05 (ns), and *P*< 0.0001 (****). (**F**, **G**) Quantification of association between RPA2 foci and pHORMAD2^S271^–rich axis segments in oocytes of 0 dpp *Spo11^–/–^* mice in the CD-1 background. Block bars show weighted averages of (F) 64.92% and (G) 7.66% from two mice; *n* = total numbers of analysed cells.

Hence, we questioned if DNA lesions that result in ssDNAs are present, and if they can potentially form the basis for HORMAD1 and HORMAD2-dependent elimination of *Spo11^–^^/^^–^* oocytes (Figure 6). We utilized either BrdU staining (Figure 6A) or co-staining of RPA2 foci with two distinct anti RPA2 antibodies (Figure 6C) to detect ssDNAs in late pachytene/diplotene oocytes. These stages are prevalent in outbred CD-1 and inbred C57Bl/6J Crl backgrounds at 0 and 1 dpp, respectively. BrdU labelling caused inviability of fetuses in C57Bl/6J Crl backgrounds. Therefore, we performed BrdU-staining only in CD-1 background; RPA2 staining was performed in both outbred (CD-1) and inbred (C57Bl/6J Crl) mice. Wild-type oocytes contained low numbers of BrdU (median, 11) and RPA2 foci (median, 4 foci for CD-1 and 10.5 for C57Bl/6J Crl) in late pachytene/early diplotene stages (Figure 6B, D, and Supplementary Figure S5A), which were characterized by fully formed axes. Once oocytes progressed to late diplotene, as identified by fragmentation of chromosome axis, recombination foci almost completely disappeared (median BrdU focus number was 1, median, RPA2 focus numbers were 0 for CD-1 and 1 for C57Bl/6J Crl, Figure 6A–D, and Supplementary Figure S5A). In contrast, hardly any BrdU and RPA2 foci were detected in *Spo11^–^^/^^–^* oocytes in stages that were equivalent to late-pachytene or diplotene based on axis morphology. As compared to wild-type, *Spo11^–^^/^^–^* oocytes had fewer BrdU (median, 1) and RPA foci (median, 2 for CD-1; 4 for C57Bl/6J Crl) in a late pachytene to early diplotene-like stage, and focus numbers were similarly low in the late diplotene-like stage (BrdU median, 1; RPA median, 0 for CD-1 and 1 for C57Bl/6J Crl, Figure 6A-D, and Supplementary Figure S5A). Accordingly, the vast majorities of pachytene-diplotene-like oocytes of *Spo11^–^^/^^–^* mice had less ssDNA foci than the 10-DSB-threshold that triggers DSB-dependent apoptosis (97%, *n* = 150 oocytes, and 96%, *n* = 145 oocytes, according to BrdU and RPA2 staining in CD-1 background, respectively, and 85%, *n* = 158 oocytes, according to RPA2 staining in C57Bl/6J Crl). In wild type, ssDNA focus numbers fell below the 10-DSB-threshold in lower fractions of pachytene-diplotene oocytes (69%, *n* = 152 oocytes, and 83%, *n* = 125 oocytes, according to BrdU and RPA2 staining in CD-1 background, respectively, and 67%, *n* = 165 oocytes, according to RPA2 staining in C57Bl/6J Crl). Oocyte quality control leads to elimination of defective oocytes primarily during or after chromosome axis disassembly according to cleaved PARP staining in wild type and *Spo11^–^^/^^–^* oocytes (Supplementary Figure S5B, C). Hence, it is unlikely that the consistently low BrdU and RPA focus numbers are the result of differential elimination of oocytes with high load of DNA damage in *Spo11^–^^/^^–^* oocytes. Together, these observations suggest that there are equivalent or reduced levels of ssDNAs in *Spo11^–^^/^^–^* oocytes as compared to wild type around birth. Therefore, persistent ssDNAs cannot explain higher levels of perinatal oocyte apoptosis in *Spo11^–^^/^^–^* mice as compared to wild type. Supporting these conclusions, RPA2 focus numbers were not elevated in *Spo11^–^^/^^–^* oocytes that were positive for the apoptosis marker cleaved-PARP as compared to oocytes that were negative (Supplementary Figure S5D). By way of exclusion, these observations suggest that asynapsis *per se*, rather than elevated numbers of unrepaired DSBs, triggers apoptosis in *Spo11^–^^/^^–^* oocytes.

### Despite diminished ssDNAs, ATR appears to be activated in perinatal *Spo11^–/–^* oocytes

Surveillance of asynapsis is thought to rely on a PI3K-like kinase, ATR, which is best known for its role in DDR (43). ATR is recruited to unsynapsed axes with the help of HORMAD1/2, which enables ATR-mediated phosphorylation of histone H2AX on serine 139 in chromatin loops that are anchored to unsynapsed axes (21,35–37). The accumulation of phospho-histone H2AX (hereafter, γH2AX) promotes transcriptional silencing of unsynapsed chromatin/MSUC (33,43,62,63). In *Spo11^–^^/^^–^* meiocytes, ATR is concentrated to an *ad hoc* subset of unsynapsed chromosomes (21,53,64) leading to the formation of well-demarcated γH2AX-rich chromatin domains. These chromatin domains are called pseudo-sex bodies (53), as their appearance, but not chromosome content, resembles the transcriptionally silenced sex body that encompasses the unsynapsed chromatin of X and Y chromosomes in spermatocytes. According to current models, perinatal deaths of *Spo11^–^^/^^–^* oocytes may be triggered by persistent ATR signalling from unsynapsed regions or silencing of essential genes within pseudo-sex bodies (21,28,35,36). Recent scientific discourse focused on the question if γH2AX-rich chromatin domains can arise independent of DSBs (see dual-checkpoint model, Figure 1B, upper panel), or if they require ssDNA lesions resulting from DSBs, as suggested by a DSB-dependent oocyte checkpoint model (Figure 1B, lower panel (28,47)). Therefore, we examined if the few RPA2 foci that were detected in some of the *Spo11^–^^/^^–^* oocytes correlated with γH2AX-rich chromatin (Supplementary Figure S5E, F). γH2AX was observed in all *Spo11^–^^/^^–^* oocytes in late pachytene and early diplotene-like stages, and it accumulated on chromatin in three distinct patterns (Supplementary Figure S5E). Most of the γH2AX-rich chromatin domains were either focal/small flares (26.25% of *n* = 739 domains, *n* = 36 cells, 2 mice) or axial (71.44% of *n* = 739 domains). A minority of γH2AX-rich chromatin domains (2.3% of *n* = 739 domains) were large, representing pseudo-sex bodies that encompassed several unsynapsed chromosome axes. Consistent with an earlier report (47), the majority (76.74%) of large γH2AX-rich chromatin domains contained RPA2 foci (Supplementary Figure S5F). However, importantly, only a small fraction (7.57%) of axial γH2AX-rich domains displayed RPA2 foci. These observations suggest that ATR signalling is active from unsynapsed chromosome axes even in the absence of ssDNAs in *Spo11^–^^/^^–^* oocytes.

To further assess if asynaptic axes promoted local ATR signalling independent of colocalizing ssDNAs we also employed an axis-restricted marker of ATR activity in oocytes. It is thought that a positive feedback drives efficient ATR activation on asynaptic chromatin. ATR phosphorylates Ser-Gln motifs within HORMAD1/2, which enhances ATR recruitment and activation by HORMAD1/2 in the context of unsynapsed axes (65). Consistent with this hypothesis, ATR, but not the paralogous ATM, is required for the accumulation of a Serine 271-phosphorylated HORMAD2 (hereafter, pHORMAD2^S271^) on unsynapsed axes of X and Y chromosomes in spermatocytes (43). Further, pHORMAD2^S271^, ATR and γH2AX jointly accumulate on asynaptic chromosomes (Supplementary Figure S6), which are present in a minority (10–15%) of wild type oocytes (40,66). In *Spo11^–^^/^^–^* oocytes, all asynaptic chromosomes domains are marked by HORMAD2, but curiously, less than half of the HORMAD2-positive axis sections are rich in ATR (Supplementary Figure S7A–C). Axial ATR, axial pHORMAD2^S271^ and axial or chromatin-wide γH2AX accumulations closely matched one another (Supplementary Figure S7D–L), indicating that ATR not only associates with a subset of unsynapsed chromosome axes in *Spo11^–^^/^^–^* oocytes, but it also is active in promoting phosphorylation of the synapsis surveillance protein HORMAD2 on axes.

Given these observations, we utilized staining of pHORMAD2^S271^ as a reporter of ATR-activity, to address if RPA2 localization correlated with ATR activity along chromosome axes in oocytes of perinatal *Spo11^–^^/^^–^* mice (Figure 6E–G). Consistent with RPA2-γH2AX comparisons, we found that whereas most RPA2 foci (64.92%, *n* = 134 foci in *n* = 38 cells) were detected in the context of pHORMAD2^S271^-positive axes (Figure 6F) only a small minority of pHORMAD2^S271^-positive chromosome axes colocalized with RPA2 foci (7.66% of pHORMAD2^S271^-positive axis domains, *n* = 1683 domains in *n* = 53 cells, Figure 6G). These observations support the conclusion that ATR signalling is prevalent from unsynapsed chromosome axes in the absence of ssDNAs in oocytes of perinatal *Spo11^–^^/^^–^* mice.

A prior study reported only few RPA foci (average 6.2) in oocytes of fetal *Spo11^–^^/^^–^* mice at a stage (17.5 dpc) where SPO11-independent DSBs were suggested to form *de novo* (47). These prior results and our data (Figure 6A–D, and Supplementary Figure S5A) suggest that DSBs are rare both in fetal and perinatal *Spo11^–^^/^^–^* oocytes. Therefore, it is unlikely that pHORMAD2^S271^-rich axial domains, which were observed in high numbers (average 32 per cell) in perinatal oocytes, mainly reflected ATR activity that was a relic of previously repaired SPO11-independent DSBs. Altogether, these observations favor the hypothesis that DSB-independent ATR signalling emerges on asynaptic chromosome regions in oocytes, and that DSB-independent ATR signalling contributes to the elimination of *Spo11^–^^/^^–^* oocytes.

### Axial ATR activity in the absence of ssDNAs in perinatal *Mcmdc2^–/–^* oocytes

**Figure 7.**
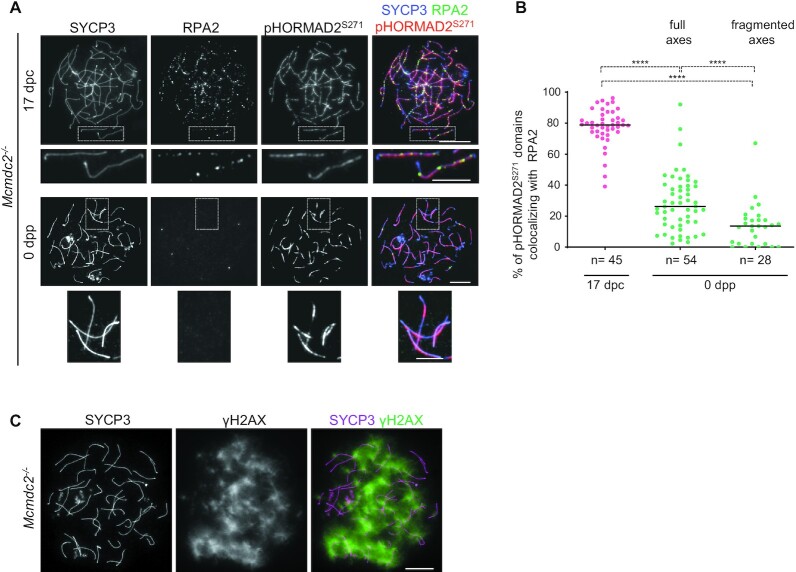
Despite diminishment of RPA2 foci, markers of ATR signalling persist till late prophase in *Mcmdc2^–/-^* oocytes. (A, C) Chromosome axis (SYCP3), and either (**A**) RPA2 and pHORMAD2^S271^ or (**C**) γH2AX were detected by immunofluorescence in surface-spread oocytes of *Mcmdc2 ^-/–^* mice either at (A) fetal 17 dpc or (A, C) 0 dpp developmental time points. (A) Enlarged insets show high pHORMAD2^S271^ levels on asynaptic axes both in the presence (at 17 dpc) and absence (at 0 dpp) of RPA2 foci. (A, C) Bars, 10 μm; in enlarged insets, 5 μm. (**B**) Quantification of pHORMAD2^S271^-rich axis domains that are associated with RPA2 in the *Mcmdc2 ^–/–^* oocytes at 17 dpc and 0 dpp time points. Quantifications are shown for oocytes with fully formed (late pachytene-early diplotene) or fragmented (late diplotene) axes at 0 dpp. Medians (bars) are 79% in 17 dpc oocytes, 26.24% and 13.62% in 0 dpp oocytes where axes are fully formed or fragmented, respectively. *n* = numbers of analysed cells from two animals. Mann–Whitney *U* test, *P*< 0.0001 (****).

Given the precedent of *Spo11^–^^/^^–^* meiocytes, we tested if pHORMAD2^S271^, as a marker of axial ATR activity, is present on asynaptic chromosomes that lack persisting ssDNA foci in DSB-proficient *Mcmdc2^–^^/^^–^* oocytes. pHORMAD2^S271^ accumulated on asynapsed axes as discontinuous domains in late zygotene or early pachytene-like oocytes that were collected from fetuses at 17 dpc. Most of these pHORMAD2^S271^-positive domains (77.4%, *n* = 4194 domains in *n* = 45 cells) associated with RPA2 foci, which were abundant at this stage (Figure 7A, B). In perinatal oocytes, most unsynapsed axes had pHORMAD2^S271^ staining, which was more intense and more continuous than at 17 dpc (Figure 7A). γH2AX-rich chromatin was also associated with most chromosome axes in perinatal oocytes (Figure 7C). In contrast, RPA2 foci associated only with a minority of pHORMAD2^S271^-positive axis domains (Figure 7B) consistent with the observation that RPA2 foci diminished in *Mcmdc2^–^^/^^–^* oocytes by birth (Figure 2C, I). Thus, ATR activity seems to persist along chromosome axes even after the turnover of most ssDNA-containing recombination intermediates in *Mcmdc2^–^^/^^–^* oocytes. It follows that asynapsis-associated ATR signalling that does not associate with persistent ssDNAs may contribute to the elimination of *Mcmdc2^–^^/^^–^* oocytes.

## DISCUSSION

Recent observations have suggested that elimination of pervasively asynaptic oocytes by the prophase checkpoint depends on high numbers of persistent DSBs in asynaptic chromosomes (28). The concept of a DSB-dependent synapsis checkpoint was supported by the observations that (1) apoptosis of the highly asynaptic *Spo11^–^^/^^–^* oocytes partially depended on the DDR kinase CHK2 (28,46), and that (2) shortly before their apoptosis in late prophase, most SPO11-deficient oocytes acquired high numbers of RAD51 foci indicative of ssDNAs resulting from DSBs (47).

Whereas foci of an alternative ssDNA marker, RPA2, were also observed in high numbers in perinatal *Spo11^–^^/–^* oocytes by a recent study (55), we detected no or very low numbers of RPA2 foci by staining *Spo11^–^^/^^–^* oocytes with two distinct RPA2 antibodies in both outbred (CD-1, Charles Rivers) and inbred (C57BL/6J Crl) backgrounds. These discrepancies may reflect distinct levels of SPO11-independent DSBs which may originate from varying transposon activity in divergent genetic backgrounds (67). Consistent with our RPA2 staining, a direct detection of ssDNAs by BrdU labelling suggests little or no ssDNAs in most *Spo11^–^^/-^*oocytes in our backgrounds. Whereas BrdU labelling is unlikely to allow efficient detection of very short ssDNA tracks, focus numbers of BrdU-labelled ssDNAs matched estimated DSB numbers in wild-type meiocytes, indicating that DNA ends are sufficiently resected to permit BrdU-based detection of most early recombination intermediates in meiosis. Further, in *Spo11^–^^/–^* oocytes, the unusually intense and extended RAD51 foci suggests long ssDNA tracks, which is expected to be particularly amenable to BrdU-based detection. Therefore, a lack of BrdU and RPA2 labelling suggests that RAD51 is not a reliable marker of ssDNAs in diplotene/dictyate oocytes.

The role of ssDNA-independent RAD51 on chromosome axis is unknown. RAD51 binds not only ssDNA but also dsDNA both *in vitro* (68) and *in vivo* in the absence of SWI2/SNF2 family DNA translocases (mammals, RAD54/RAD54B (60) and budding yeast, Rdh54 (69)). Given these precedents, axis-association of RAD51 in *Spo11^–^^/–^* oocytes in late prophase may merely indicate that asynaptic axes provide a permissive environment for RAD51 accumulation on undamaged dsDNAs. Alternatively, RAD51 might directly bind to axis components or associated proteins without interacting with DNA. Asynaptic axes acquire filamentous RAD51, ATR and ATR co-factors, including BRCA1 and TOPBP1, on a HORMAD1/2-dependent manner in meiocytes (21,28,35,36,38). Therefore, ssDNA-independent recruitment of RAD51 to axes might be promoted by HORMAD1/2 or dependent proteins functioning in ATR signalling. In particular, BRCA1 may be involved as RAD51 and BRCA1 form soluble complexes in mammalian cells (70).

Most S*po11^–^^/–^* oocytes acquire very few if any ssDNA foci whose numbers are well below the 10-DSB-threshold that effectively induce apoptosis in wild-type oocytes. Therefore, our data provides evidence for a synapsis checkpoint mechanism that does not obligately depend on high numbers of persistent ssDNAs in line with the dual prophase checkpoint model (Figure 1B, upper panel). Prior data suggest that HORMAD1/2-dependent recruitment of ATR activity to unsynapsed chromosome axes leads to apoptosis of persistently asynaptic oocytes in the absence of SPO11-dependent DSBs (21,35,37). During DDR, ATR activation requires (1) a recruitment of ATR-ATRIP complexes to RPA coated ssDNAs and (2) a recruitment of the ATR-activator TOPBP1 to ssDNA-dsDNA junctures (71,72). Importantly, ATR is activated in the absence of DNA damage by optogenetic induction of TOPBP1 condensation, which, as part of a positive feedback, requires TOPBP1 phosphorylation by basal ATR activity *in vivo* (73). Thus, molecular crowding and positive feedback of ATR and its auxiliary factors are sufficient to drive ATR activation. ATR, BRCA1 and TOPBP1 are interdependent for axial accumulation (43,74,75), contrasting ATR binding to ssDNAs, which is independent of TOPBP1 (76). These observations suggest that positive feedbacks drive activation of ATR on unsynapsed axes. Whereas the exact molecular mechanism is not known, we propose that axis-bound HORMAD1/2 provides an anchor for ATR and/or its auxiliary factors thereby enabling ATR activation on unsynapsed axes by molecular crowding even in the absence of DSBs. ATR phosphorylates both HORMAD1^Ser374^ and HORMAD2^Ser271^, which was proposed to enhance axial ATR recruitment thereby solidifying ATR signalling on asynaptic chromosome axes (43,65). An additional positive feedback involving the ATR-phospho target histone γH2AX and MDC1 promotes spreading of ATR activity to axis-associated DNA loops, further amplifying ATR signalling in unsynapsed regions (77).

Whereas our data suggest that axial ATR activation underlies a synapsis checkpoint that is distinct from the checkpoint that monitors unrepaired DSBs (dual checkpoint model, Figure 1B, upper panel) it is likely that there is crosstalk between these two checkpoint pathways. We speculate that unrepaired DSBs acquire ATR that phosphorylates HORMAD1/2 at nearby axial sites, which may efficiently seed ATR recruitment to asynaptic axes. Consistent with this hypothesis and in line with an earlier report (47), we found that ATR-rich pseudo sex bodies often (76%) contain RPA2 foci that likely represent spontaneously occurring DSBs in *Spo11^–^^/^^–^* oocytes. Thus, while ATR is activated on a considerable fraction of unsynapsed regions without DSBs, if DSBs occur, they efficiently drive ATR build-up in their vicinity, leading to the formation of extended chromatin domains of high ATR activity.

Asynapsis-associated ATR may trigger apoptosis of *Spo11^–^^/^^–^* oocytes by (i) promoting MSUC and resultant repression of essential genes in affected chromatin and/or (ii) silencing-independent ATR signalling. In DSB-proficient backgrounds, MSUC is effective only if asynapsis is limited. It is thought that if asynapsis is extensive, ATR signalling/silencing factors cannot reach densities needed for efficient MSUC because ATR signalling/silencing factors are distributed to large numbers of DSBs and associated sections of axes (40,42). We found that the distribution of ATR signalling/silencing factors is uneven on unsynapsed chromatin in the absence of SPO11-dependent DSBs, as evidenced by restriction of ATR accumulation to less than half of unsynapsed regions in *Spo11^–^^/–^* oocytes. We attribute uneven ATR accumulation to the combination of (i) absence or low numbers of DSBs, (ii) inefficient ATR seeding in asynaptic regions that lack DSBs and (iii) a positive feedback that supports efficient spreading of ATR activity around both DSB-dependent and –independent seeding sites. Whereas the large majority of axis sections that acquire ATR activity do not contribute to pseudo sex bodies, a considerable fraction of *Spo11^–^^/–^* oocytes (47.2% in this study) form pseudo sex bodies. The chromatin is silenced in pseudo sex bodies of *Spo11^–^^/^^–^* spermatocytes (42). Whereas MSUC is less efficient in oocytes than spermatocytes (41), concentrated ATR signalling may sufficiently disrupt transcription of essential genes in asynaptic regions to trigger apoptosis in *Spo11^–^^/–^* oocytes.

Alternatively, asynapsis-induced ATR activity may promote apoptosis by (i) directly phosphorylating and activating pro-apoptotic transcription factors (e.g. TRP53 phosphorylation on Ser18, equivalent to Ser15 in human (78–80)), or (ii) activating DDR signalling (reviewed in (81)). Loss of CHK2 DDR kinase reduces apoptosis of *Spo11^–^^/–^* oocytes by ∼35%, which may indicate that rare SPO11-independent DSBs lead to CHK2 activation thereby contributing to elimination of *Spo11^–^^/–^* oocytes (28,46). The number of DSBs in *Spo11^–^^/^^–^* rarely (5–15%) exceeded the reported lethal dose of DSBs in wild type. Hence, we speculate that DSBs induce apoptosis more efficiently in *Spo11^–^^/^^–^* than wild type, or CHK2 may also be activated by axis-associated ATR signalling independent of DSBs. DNA damage-independent TOPBP1 condensate formation does not only activate ATR, but also leads to increased activity of the downstream DDR kinase, CHK1, in somatic cells (73). Given this precedent, molecular crowding of ATR signalling factors may activate downstream DDR signalling on asynaptic axes in the absence of DSBs. Whereas it has been difficult to definitively test the role of CHK1 during oogenesis due to embryonic lethality of *Chk1^–^^/–^* mice (82), CHK1 complements CHK2 in the induction of perinatal apoptosis in DSB-proficient oocytes (46). Hence, CHK1 may also complement CHK2 in triggering apoptosis of *Spo11^–^^/–^* oocytes. Simultaneous inactivation of both CHK1 and CHK2 will be necessary to test if DDR signalling induces apoptosis of most *Spo11^–^^/^^–^* oocytes.

Elimination of both *Spo11^–^^/–^* and DSB repair defective oocytes (e.g. *Trip13^–^^/–^* and *Dmc1^–^^/^^–^*) involves DDR signalling and a downstream activation of proapoptotic transcription factors, TRP53 and TAP63 (28,39,45). Yet, the effector pathways of apoptosis appear distinct in *Spo11^–^^/–^* and DSB repair-defective oocytes; whereas apoptosis of DSB repair-defective oocytes (*Msh5^–^^/^^–^* and *Dmc1^–^^/^^–^*) depends on BCL-2 pathway proteins, PUMA, NOXA and BAX, apoptosis of asynaptic *Spo11^–^^/^^–^* oocytes does not (44). A straightforward interpretation is that distinct defects trigger apoptosis in *Spo11^–^^/^^–^* and DSB repair-defective oocytes, which is consistent with the hypothesis of a synapsis checkpoint that does not require persistent DSBs above wild-type levels.

Loss of HORMAD1/2 caused a reduction of endogenous RAD51 foci and increased turnover of irradiation-induced recombination foci in *Spo11^–^^/-^* meiocytes (28,29). These observations gave rise to the hypothesis that HORMAD1/2 enabled checkpoint activation by delaying DSB repair leading to DDR-mediated apoptosis in oocytes. However, in these experiments, either only the turnover of RAD51 foci was examined (28), or, where DMC1 and RPA were also examined (29), the turnover of DMC1 foci was only modestly increased, and RPA foci were not affected by HORMAD1 loss. We found that RAD51 foci do not reliably mark ssDNAs in oocytes, which thus questions if and to what extent HORMAD1/2 delays DSB repair on unsynapsed axes. Significantly, DSB repair is not indefinitely delayed if asynapsis was caused by chromosomal abnormalities (33) or a deficiency of the recombination protein MCMDC2 (this study) in oocytes. In both types of models, DSB foci disappear from asynaptic axes once oocytes progress to late pachytene and diplotene. These observations mirror the turnover of recombination foci on unsynapsed XY chromosomes in late pachytene, which is thought to reflect enablement of DSB repair by inter-sister recombination or NHEJ in late prophase (12). In all these cases, HORMAD1/2 persist on unsynapsed axes indicating that HORMAD1/2 are unable to efficiently block DSB repair in late prophase in both sexes. Hence, we favor the idea that HORMAD1/2 function in meiotic prophase checkpoints primarily entails amplifying and maintaining ATR signalling from asynaptic axes as opposed to preventing repair of DSBs. Beyond permitting elimination of DSB-deficient oocytes, this HORMAD1/2-mediated ATR signalling likely aids quality control of DSB-proficient oocytes too. In oocytes where key recombination proteins are functional, most DSBs are repaired, and whether or not asynapsis abnormally occurred, most chromosomes are unsynapsed by late diplotene. HORMAD1/2-mediated maintenance of ATR signalling on asynaptic axes provides a memory of failed homolog synapsis in the absence of persistent ssDNAs, which may enable delayed elimination of asynaptic oocytes by apoptotic pathways that are primarily activated in late prophase.

## DATA AVAILABILITY

The data supporting the findings of this study are available within the paper. The source data underlying both main and supplementary figures are provided as a Source Data file.

## Supplementary Material

gkac355_Supplemental_Files
