# Supplementary material for: Chromosomal synapsis defects can trigger oocyte apoptosis without elevating numbers of persistent DNA breaks above wild-type levels
Source: Nucleic Acids Res. 2022 May 17;50(10):5617–34. doi: 10.1093/nar/gkac355 (PMC9177993; doi:10.1093/nar/gkac355)
Supplement: gkac355_Supplemental_Files [file gkac355_supplemental_files.zip › Manuscript_Ravindranathan_Toth_SupplementaryData_revised.pdf]

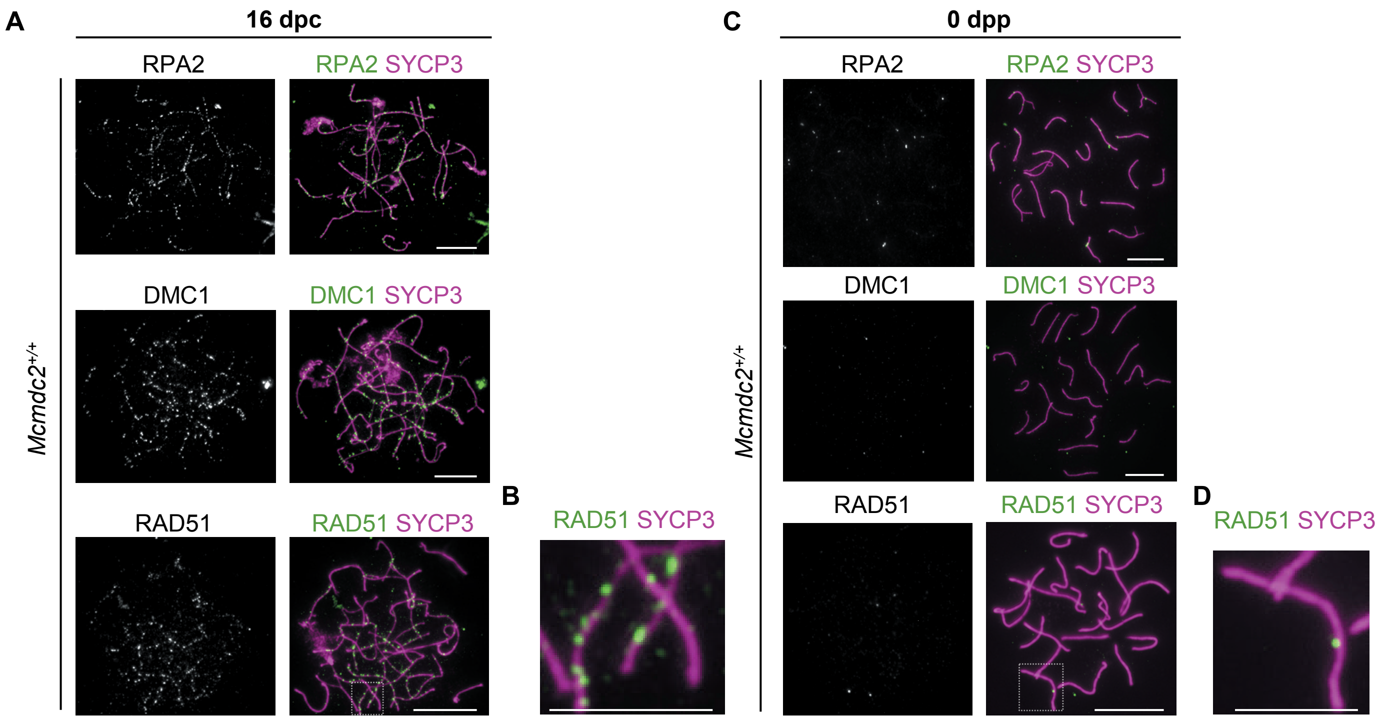

Supplementary Figure S1. Recombination foci diminish in wild-type oocytes by birth
(A, B, C, D) Chromosome axis (SYCP3) and recombination proteins RPA2, DMC1 or RAD51 were detected by immunofluorescence in surface-spread oocytes of **(A, B)** 16 dpc fetuses and **(C, D)** newborn mice (0 dpp) in wild type. **(B, D)** Enlarged insets show RAD51 foci on axes. Bars, 10 µm; in enlarged insets, 5 µm.

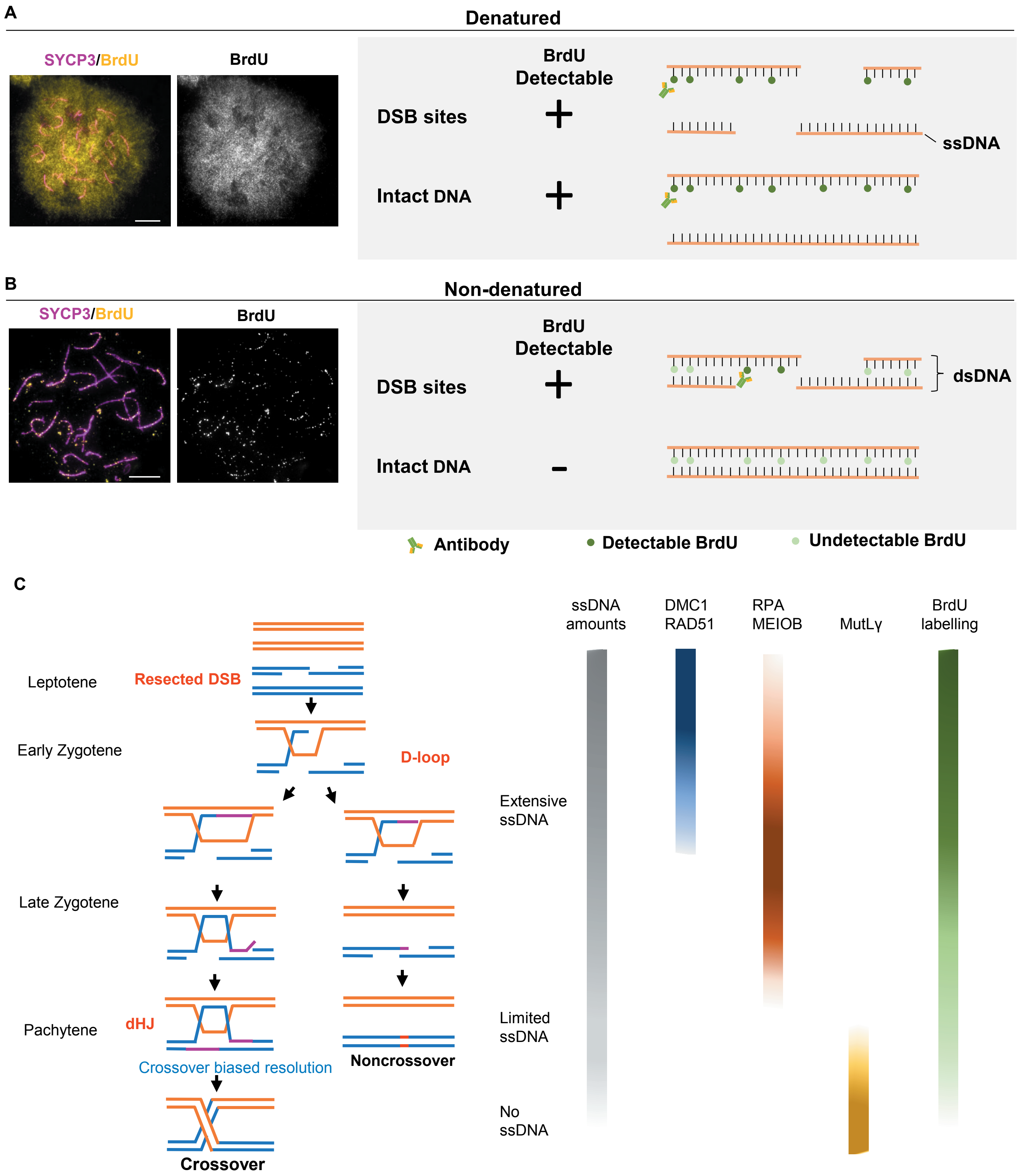

Supplementary Figure S2. BrdU labelling of recombination–associated ssDNA in meiocytes

(A left, B left) Chromosome axis (SYCP3) and BrdU were detected by immunofluorescence in surface-spread oocytes of wildtype 16 dpc fetuses either **(A)** after or **(B)** without denaturation. **(A right, B right)** Schematics shows that BrdU antibody detects BrdU genome-wide or only in the context of resected ssDNA-ends **(A)** after or **(B)** without denaturation, respectively. Bars, 10 µm. **(C)** Schematics show key steps of meiotic recombination indicating the stages of prophase and the presence of ssDNAs, key recombination proteins (DMC1/RAD51, RPA/MEIOB, MutLγ) and BrdU. In the schematics on the left, blue and orange lines indicate strands of DNA in homologous chromosomes, magenta indicates newly synthesised DNA strands.

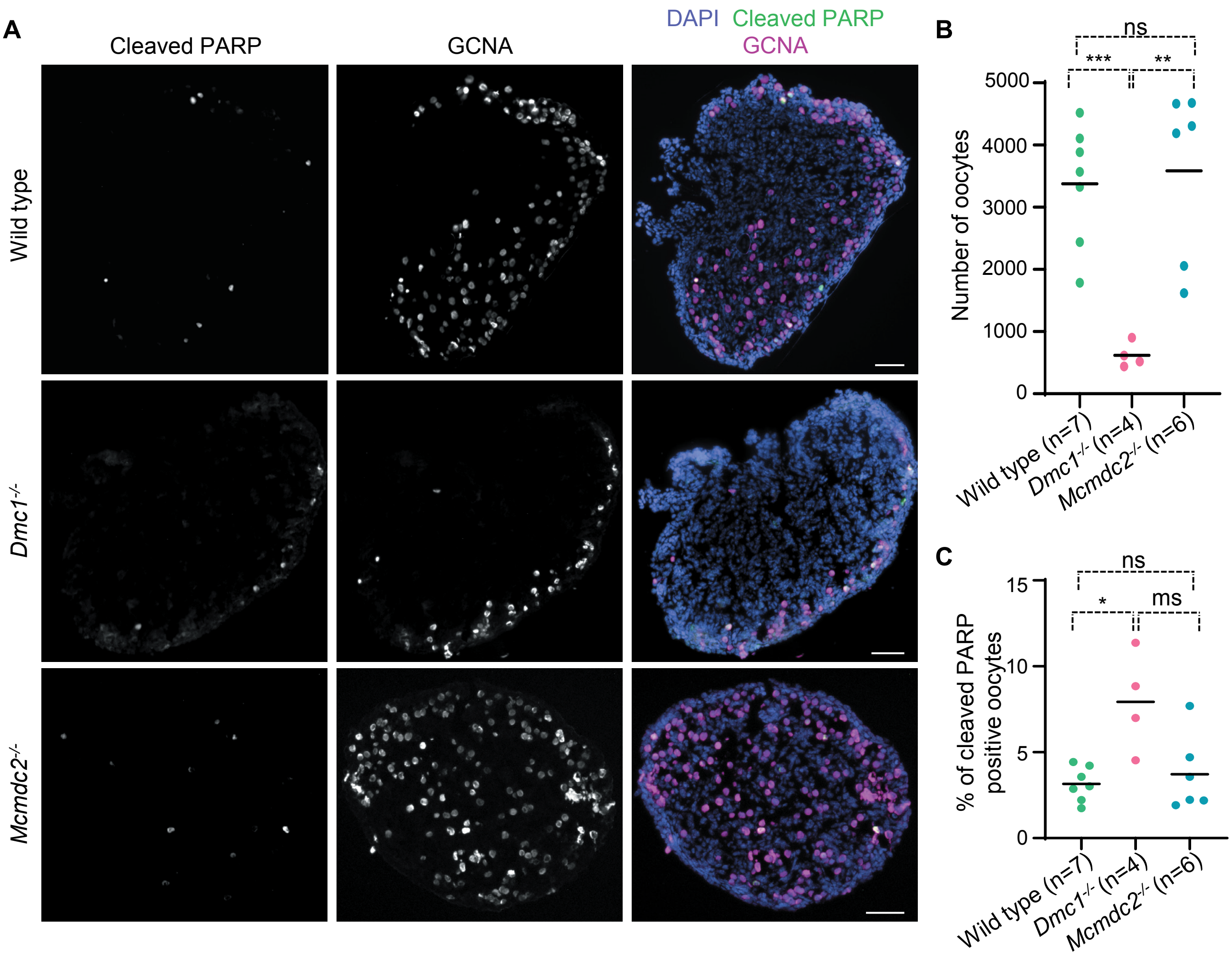

Supplementary Figure S3. Oocyte apoptosis is earlier and less efficient in *Mcmdc2$^{-/-}$* than *Dmc1$^{-/-}$* mice

(A) DNA, an oocyte marker (GCNA) and a marker of apoptosis (cleaved PARP) were detected in sections of ovaries of newborn wild-type, *Dmc1$^{-/-}$* and *Mcmdc2$^{-/-}$* mice. Bars, 50 µm. **(B, C)** Quantification of **(B)** oocyte numbers (GCNA) or **(C)** the proportions of apoptotic (cleaved-PARP positive) oocytes in sections of ovaries of newborn mice. Ovaries were sectioned and oocytes with or without cleaved-PARP staining were counted in every 7th section. Data points represent combined counts from the two ovaries of each mouse. Averages (bars) are **(B)** 3376 in wild type, 620 in *Dmc1$^{-/-}$* and 3585 in *Mcmdc2$^{-/-}$* mice, **(C)** 3.15% in wild-type, 7.93% in *Dmc1$^{-/-}$* and 3.72% in *Mcmdc2$^{-/-}$* mice. Two tailed t test with Welch correction, non-significant P>0.1 (ns), marginally significant P=0.0533 (ms), 0.05>P>0.01 (*), P>0.001 (**), P>0.0001 (***). n= number of animals analyzed.

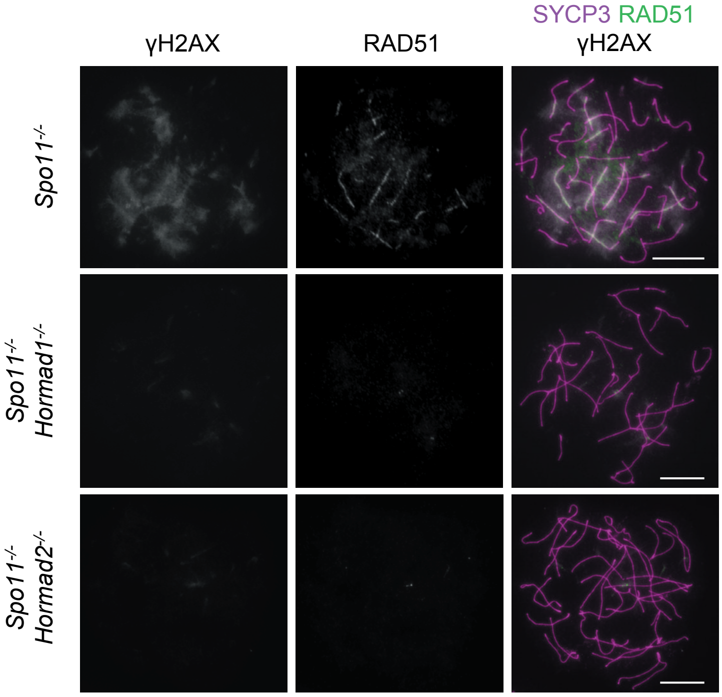

Supplementary Figure S4. HORMAD1 is required for filamentous RAD51 accumulations on axes of asynaptic *Spo11$^{-/-}$* oocytes. Chromosome axis (SYCP3), RAD51 and γH2AX were detected by immunofluorescence in surface-spread oocytes of *Spo11$^{-/-}$* , *Spo11$^{-/-}$ Hormad1$^{-/-}$* and *Spo11$^{-/-}$ Hormad2$^{-/-}$* mice (C57BL/6J background) one day after birth. Bars, 10 µm.

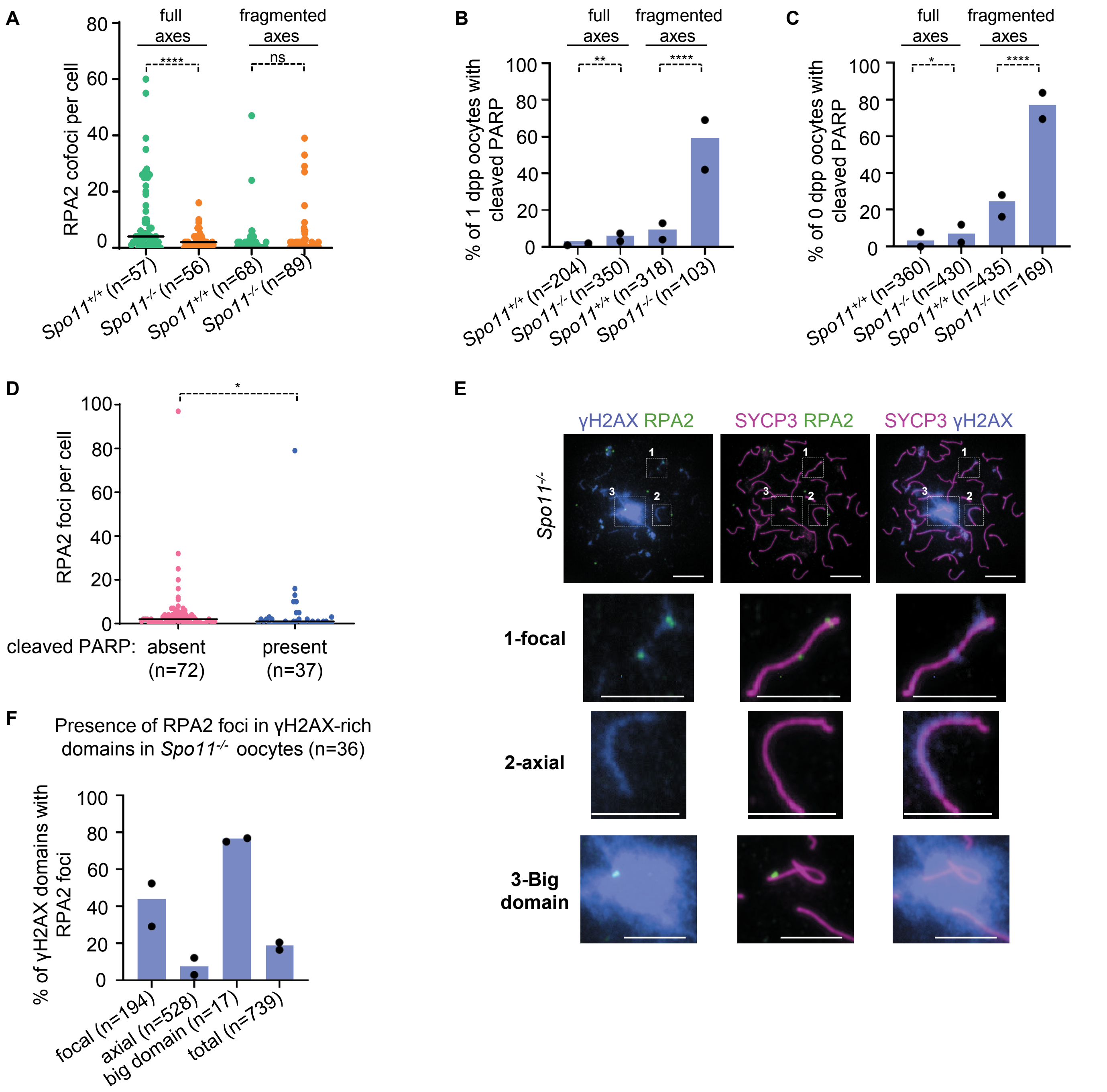

Supplementary Figure S5. Markers of apoptosis, ssDNA and ATR activity in *Spo11$^{-/-}$* oocytes around birth

(A) Quantification of axis-associated RPA2 cofoci, i.e. foci simultaneously detected by rat and rabbit anti-RPA2 antibodies, in the oocytes of 0 dpp mice of the indicated genotypes in a CD-1 background. Focus counts are shown in oocytes where axis was either fully formed (late pachytene-early diplotene) or fragmented (late diplotene). Medians (bars) are 4, 0, 2 and 0 from left to right. **(B, C)** Proportions of apoptotic (cleaved PARP positive) oocytes in *Spo11$^{+/+}$* and *Spo11$^{-/-}$* mice at 1 dpp (**B**, C57BL/6J background) or 0 dpp (**C**, CD-1 background). Analysed oocytes had either fully formed or fragmented axes. n=oocyte numbers analysed from two mice; weighted averages (block bars) are 1.5, 9.43, 6.29 and 59.2 in **B** and 3.33, 24.6, 6.97 and 76.92 in **C** from left to right. **(D)** Graph shows RPA2 focus numbers in *Spo11$^{-/-}$* oocytes where cleaved PARP was absent or present at 0 dpp (early broken axis stage). Medians (bars) are 2 in PARP negative and 1 in PARP positive oocytes. **(E)** Images show SYCP3, γH2AX and RPA in oocytes of 0 dpp *Spo11$^{-/-}$* mice (CD-1 background). Enlarged insets show distinct categories of γH2AX localization patterns in *Spo11$^{-/-}$* oocytes at 0 dpp. Bars, 10 µm; in enlarged insets, 5 µm. **(F)** Quantification of the presence of RPA2 foci in γH2AX-rich chromatin domain sub-types that are ilustrated in **E**. n=numbers of analysed γH2AX-rich chromatin domains from two mice; weighted averages (block bars) are 43.81% in focal , 7.57% in axial, 76.47% in big domain and 18.67% in combined (total) categories of γH2AX-rich domains. **(A, D)** Mann–Whitney U test and **(B, C)** a likelihood ratio test, non-significant P>0.05 (ns), P>0.01 (*), P>0.001 (**) and P<0.0001 (****)..

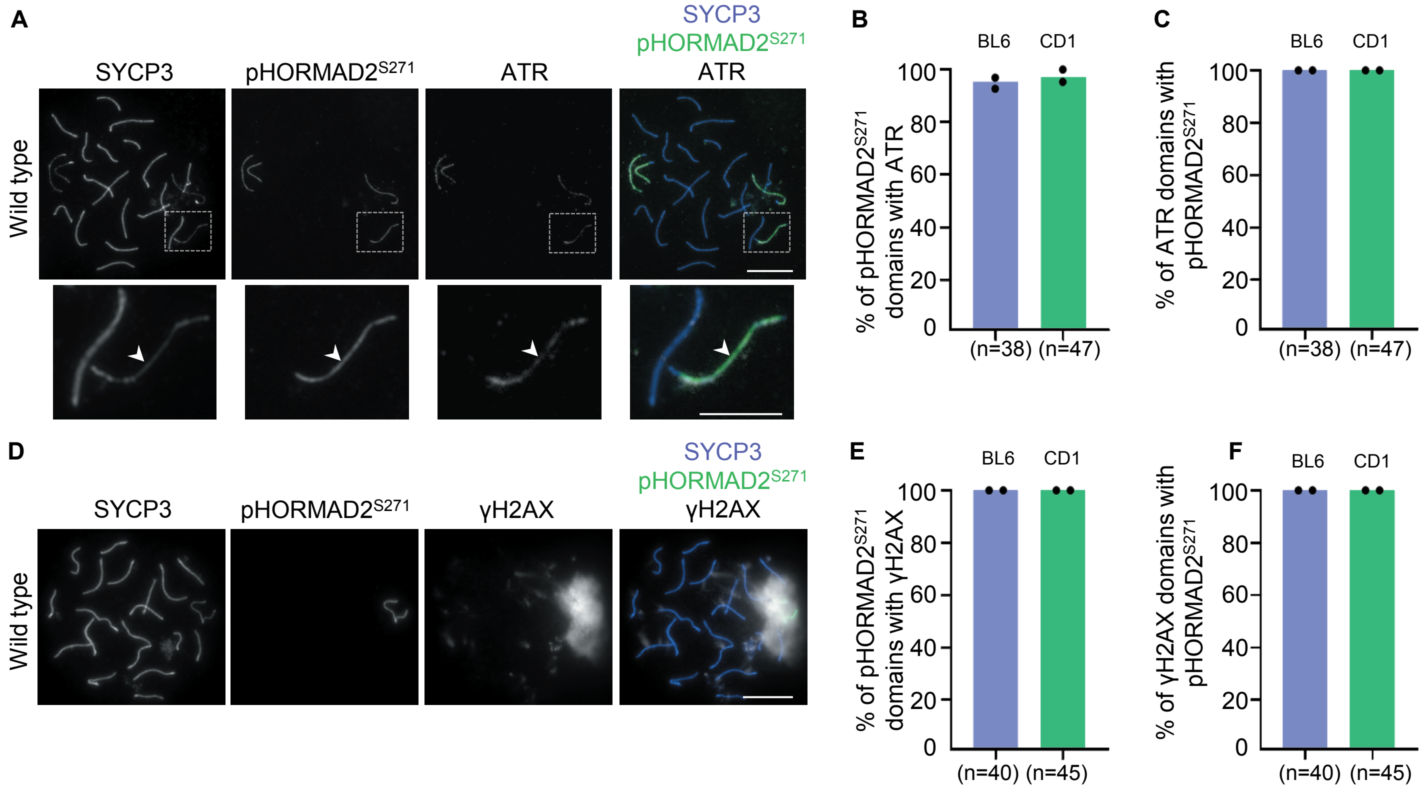

Supplementary Figure S6. Axial pHORMAD2^{S271} correlates with ATR and γH2AX accumulation on asynaptic chromatin

(A) Chromosome axis (SYCP3), pHORMAD2^{S271} and ATR were detected by immunofluorescence in surface-spread oocytes of newborn (0 dpp) wild-type mice. Enlarged insets in **A** show correlated accumulation of pHORMAD2^{S271} and ATR on an asynaptic chromosome (arrowhead). **(B, C)** Quantification of correlation between the accumulation of axial pHORMAD2^{S271} and axial ATR. n=number of asynaptic oocytes analysed from two mice; weighted averages (block bars) are **(B)** 95.5% in C57BL/6J, 97.35% in CD-1 and **(C)** 100% in both C57BL/6J and CD-1 mice. **(D)** Immunofluorescence staining of a surface-spread oocyte of newborn wild-type mouse shows accumulation of pHORMAD2^{S271} and γH2AX on the axis and chromatin of an asynaptic chromosome, respectively. **(E, F)** Quantification of correlation between the axial pHORMAD2^{S271} and big γH2AX-rich chromatin domains in asynaptic oocytes of newborn mice. n=number of asynaptic oocytes analysed from two mice; weighted averages (block bars) are 100% in both C57BL/6J and CD-1 mice. Bars, 10 µm; in enlarged insets, 5 µm.

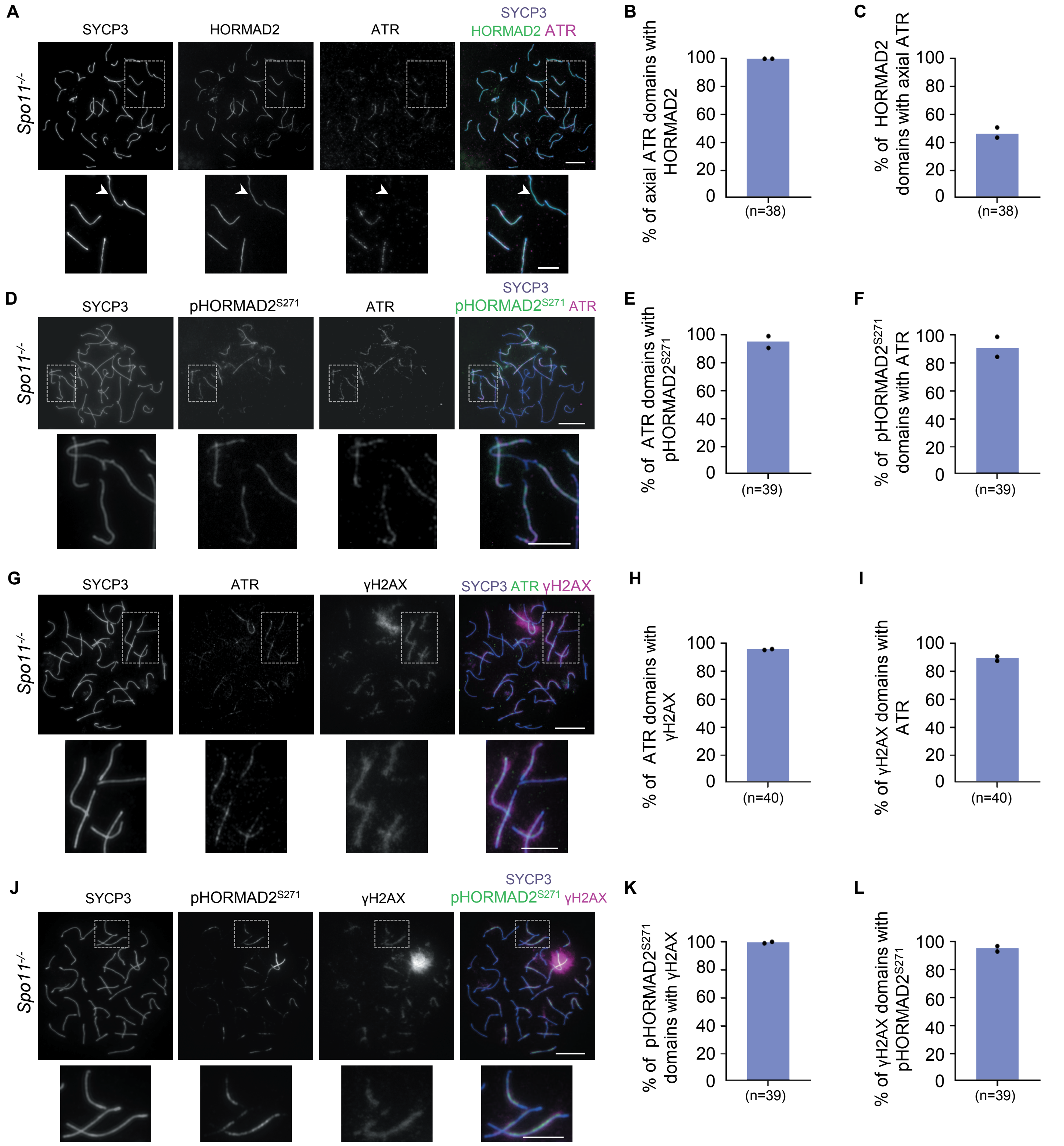

Supplementary Figure S7. Accumulation of axial ATR, axial pHORMAD2^{S271} and chromatin-bound γH2AX correlate on asynaptic chromosomes of *Spo11$^{-/-}$* oocytes.

(A, D, G, J) Indicated proteins were detected in surface-spread oocytes of newborn (0 dpp) *Spo11$^{-/-}$* mice by immunofluorescence. Enlarged insets show images of asynaptic chromosomes. **(A)** Arrowhead point at a HORMAD2-marked unsynapsed axis where ATR did not accumulate. Bars, 10 µm; in enlarged insets, 5 µm. Quantification of correlations between (**B**, **C**) HORMAD2 and axial ATR, (**E**, **F**) pHORMAD2^{S271} and ATR, (**H**, **I**) ATR and γH2AX and (**K**, **L**) pHORMAD2^{S271} and γH2AX on asynaptic chromosomes in oocytes of newborn (0 dpp) *Spo11$^{-/-}$* mice (CD-1 background). n=number of asynaptic oocytes analysed from two mice; weighted averages (block bars) are **(B)** 99.71%, **(C)** 46.43%, **(E)** 95.19%, **(F)** 90.36%, **(H)** 95.59%, **(I)** 89.36%, **(K)** 99.53%, **(L)** 95.02%.

Recombination marker	Reliable detection after treatment with trypsin and pepsin
**Early recombination markers:**	
DMC1	NO
RAD51	NO
**Late recombination markers:**	
RPA	NO
MEIOB	YES
**Cross over markers:**	
MLH1	NO
PRR19	NO
CNTD1	YES
**Axes markers:**	
SYCP3	YES
HORMAD2	YES
Phospho HORMAD2	YES
SYCP1	NO

Table S1

The table summarizes how reliable the detection of meiotic recombination/chromosomal proteins is after chomosome spreads are treated by trypsin and pepsin to allow BrdU detection.

Supplementary Methods

Analysis of deviance using the likelihood-ratio test based on the chi-squared distribution (used in Supplementary Fig. S5B and S5C):

```
## Download the R-package which can be used to fit models with random effects:
library(lmerTest)
library(xlsx)
## Download the data
dat <- read.xlsx("your file name.xlsx",1)
##below is example table format
my.data.example <- data.frame(expand.grid(
  Level = c("a","b" ,"c"),
  Experiment=c("1","2"),
  Type = c("wt","ko"))  )

my.data.example$Y1 <-c(155, 79,11,143,52,65,16,206,55,222,19,91)
my.data.example

## Look at the data:
dat
## The analysis of the data in the column Y1 is now explained in detail:
## In order to test the interaction between Type and Level,
## two models are fit. In the first model, the interaction is present:
model.1.1 <- glmer(Y1 ~ Type + Level + Type*Level + (1|Experiment),family =
poisson, data=dat)
## To see the summary of the model fit:
summary(model.1.1)
## In the second model, the interaction term is not included
model.1.2 <- glmer(Y1 ~ Type + Level + (1|Experiment),family = poisson, data=dat)
## To see the summary of the model fit:
summary(model.1.2)
## The two models are now compared using the anova() command:
anova(model.1.1,model.1.2)
## This command compares the model fit using the likelihood-ratio test.
## Because the p-value of the likelihood ratio test is less than 5%, we
## conclude that the data provide evidence that the interaction is significant.
## Therefore, we have evidence rgar the distibutions of Level are different
## between the two types (wt and ko).
```
